# Fast adaptation to rule switching using neuronal surprise

**DOI:** 10.1371/journal.pcbi.1011839

**Published:** 2024-02-20

**Authors:** Martin L. L. R. Barry, Wulfram Gerstner

**Affiliations:** School of Computer and Communication Sciences and School of Life Sciences, Ecole Polytechnique Fédérale de Lausanne, Lausanne, Switzerland; Research Center Jülich, GERMANY

## Abstract

In humans and animals, surprise is a physiological reaction to an unexpected event, but how surprise can be linked to plausible models of neuronal activity is an open problem. We propose a self-supervised spiking neural network model where a surprise signal is extracted from an increase in neural activity after an imbalance of excitation and inhibition. The surprise signal modulates synaptic plasticity via a three-factor learning rule which increases plasticity at moments of surprise. The surprise signal remains small when transitions between sensory events follow a previously learned rule but increases immediately after rule switching. In a spiking network with several modules, previously learned rules are protected against overwriting, as long as the number of modules is larger than the total number of rules—making a step towards solving the stability-plasticity dilemma in neuroscience. Our model relates the subjective notion of surprise to specific predictions on the circuit level.

## Author summary

Everybody knows the subjective feeling of surprise and behavioral reactions to surprising events such as startle response and pupil dilation are widely studied—but how can surprise arise from neural activity? And why is surprise useful? To answer these questions we use a modeling approach. We design a self-supervised spiking neural network capable of extracting surprising information from its own activity. Surprise is measured by a mismatch between the representation of the current stimulus inside the model and the expectations of the model given previous stimuli. We propose a specific network architecture which allows the network—in combination with a three-factor NeoHebbian learning rule—to detect rule changes, signal these changes as a surprise signal, and in turn use the surprise signal to rapidly re-adapt the model’s predictions of possible next stimuli. Our bottom-up model presents a concrete hypothesis of a bio-plausible implementation of surprise and makes several specific experimental predictions for future in vivo studies.

## Introduction

An event is surprising if it does not match our expectations [1–4]. The unexpected punchline of a joke [3], the unexpected continuation of a sequence of tones [5], harmonies [6, 7] or images [8–10], as well as rule switching such as shift of escape in the Morris watermaze [11] or meaning of cues [12–15] induce measurable physiological and behavioral reactions in humans and animals. Without expectations arising from previous experiences, an event such as the observation of a new image may be perceived as ‘novel’ but cannot be ‘surprising’ [16, 17].

Surprise is a well-studied phenomenon in the neurosciences [1, 2] and has also been formally analyzed in the mathematical literature [4]. In the neurosciences, startle responses [18], delayed responses [2] and pupil dilation [19, 20] are measurable physiological manifestations in response to surprising events. Moreover, EEG, fMRI, MEG, and electrophysiological studies show an increase in brain activity shortly after a surprising event [1, 9, 21–25]. Apart from its potential role for intrinsic motivation [26], surprise plays a crucial role in learning: surprising events are more memorable [2, 27, 28] and allow quick adaptation to a changing environment [29, 30]. In this modeling paper, we study the role of surprise in building expectations, modulating learning, and detecting rule switches. Specifically, we focus on two aspects. First, surprising events significantly increase the speed of learning [16, 31–33] presumably by increasing synaptic plasticity. Second, surprise is involved in the creation and consolidation of memories [2, 34, 35], presumably including the memory of rules.

In contrast to mathematical studies that start from a normative framework of surprise [4, 36–46], we take a constructive approach based on a network of spiking model neurons with plastic connections. We consider two aspects of spiking neural networks as crucial requirements for biological plausibility. First, all information about expected and observed events, and an occasional mismatch between the two, needs to be *communicated via spikes*; thus a comparison of subthreshold membrane potentials across different neurons—as required in some existing models [47–49]—is not possible. Second, synaptic plasticity rules should be expressed as *NeoHebbian three-factor learning rules* [50–55] where the changes of a synapse from neuron A to neuron B can only depend on the spikes of neuron A and the state of neuron B (the two ‘local’ factors’) plus one (or several) neuromodulators that play the role of a global feedback signal (third factor) broadcast to large groups of neurons; in our approach, detailed synapse-specific feedback as used in the BackProp algorithm [56] and variants thereof [57–60] is not needed.

Our main assumption is that surprise manifests itself in a spiking neural network as a *mismatch between excitation and inhibition* in a layer of hidden neurons that represent the current observation and compare it to the expectation arising from earlier observations. Our approach is intimately linked to both the theory of excitation-inhibition balance (E-I balance) [61–63] and the theory of predictive coding [64–67].

Predictive coding is an influential theory in the fields of neurosciences [24, 64, 68–70] and bio-inspired artificial neural networks [71–74]. In contrast to the classic framework of predictive coding that emphasizes sparsity of activity as a means to minimize redundancy of codes [75], we emphasize the advantage of predictive codes for generating a surprise signal in spiking neural networks. Importantly, we propose in this paper that *an intrinsic spike-based surprise signal can modulate biologically plausible synaptic plasticity rules so as to achieve fast adaptation and continual learning across rule switches*.

Prediction errors in predictive coding are typically local, e.g., if a subject tries to predict the next image in a sequence, each wrong pixel gives a local prediction error. Similarly, E-I balance is often defined on a per-neuron basis, i.e., each neuron tries to balance excitatory and inhibitory inputs (detailed balance) [63]. In contrast to local prediction errors and detailed E-I balance, we consider in this paper surprise as a more global signal that sums over many local prediction errors, or many E-I mismatch signals, to extract an area-wide surprise signal that can be classified as an observation-mismatch surprise [4]. We emphasize that surprise is not necessarily conscious. Indeed, unexpected continuations of tone sequences or harmonies evoke EEG signals even in subjects without musical education [5, 7].

We focus on two related tasks both involving sequences of observations. The first task illustrates the well-known problem of re-adaptation to abrupt switches in the stimulus statistics where the same rule of stimulus generation is unlikely to occur twice [31, 42, 46]; the second one exemplifies the problem of continual learning across rule switches where each rule should be memorized since it is likely to re-appear [11, 15, 76]. In both tasks, expectations (‘predictions’) must be built by self-supervised learning, and change points (‘rule switches’) must be inferred from the observation sequence since they are not indicated by a cue. Our model links observations in the neurosciences at the level of single neurons or circuits to psychological phenomena of surprise and provides an alternative to algorithmic approaches to the stability-plasticity dilemma [77, 78], continual learning [13, 76, 79, 80], context-dependent prediction [81–83], or context buffers in artificial neural networks [72].

## Results

### Building expectations in a sequence task with rule switching

Imagine the following sequence of numbers

$$\begin{array}{c} \underset{\text{rule}\;A}{\underset{︸}{1\to 2\to 3\to 4\to 1\to 2\to 3\to 4}}\to \underset{\text{rule}\;B}{\underset{︸}{2\to 1\to 4\to 3\to 2\to 1\to 4\to 3}}\;\;. \end{array}$$ (1)

The complete sequence is composed of **transitions** (e.g. 2 → 3) and **switches** between two different rules (rule *A* → rule *B*). We refer to the moment of rule switching as a change point. The “volatile sequence tasks” used in this paper generalize switches between elementary deterministic rules, as in (1), to more complex probabilistic sequences of images generated by the following procedure. We have a total of R images. After presentation of image number *i*, the next image is one of *K* images that are possible as successors of *i*. For example, for *K* = 2 the possible successors after the image ‘apple’ could be ‘pen’ or ‘hat’ with equal probability. We will see later that, when the rule of sequence generation changes (e.g., ‘apple’ is followed by ‘car’), participants watching the sequence of images report the subjective feeling of surprise, consistent with earlier experiments [84, 85]. Thus in such a framework, a change point triggers a surprise signal.

**Fig 1:**
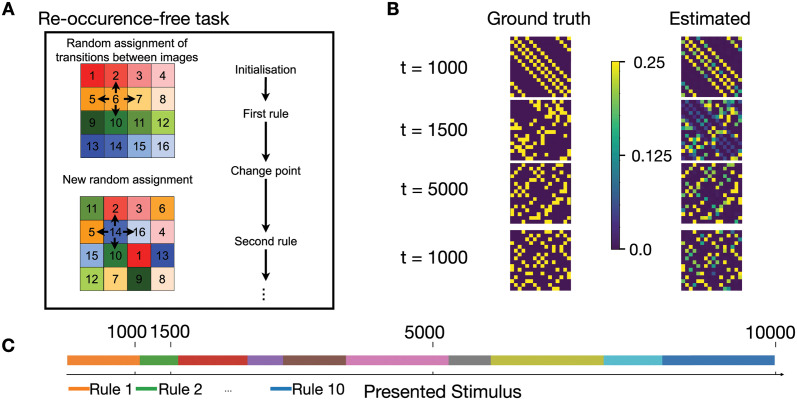
Expected transitions in a volatile sequence task. **A**. At each presentation step, the stimulus presents the wallpaper image (indicated by different colors) in one of the rooms of an apartment with R rooms (here R=16). The stimulation sequence reflects transitions (arrows) from the current room (current image) to one of the *K* neighboring rooms (here *K* = 4). On rare occasions (change points), the transition rule is changed by a new random assignment of images to rooms. The same rule is unlikely to return. **B**. The ground truth transition matrix $T_{i,j}^{*}\left(m\right)$ for different rules *m* = 1, … 4 (left, yellow indicates $T_{i,j}^{*}=1/4$, dark blue $T_{i,j}^{*}=0$), compared to the transition matrix *T*_*ij*_ estimated by the model (right, light blue and green: 0 < *T*_*ij*_ < 1/4) at different time points of a simulation run. Rule 1 at *t* = 1000 corresponds to the first configuration in A. **C**. Switching of rules over time in the simulation of B. Each rule (Rule 1, Rule 2, …) only appears once. Vertical lines indicate the time points in B.

To generalize the above procedure to the case of *K* = 4 possible successor images, we may think of a video taken in an empty apartment of R square rooms, each room recognizable by a specific wallpaper. The video camera takes one static image of a room before it is moved to one of the *K* neighboring rooms (Fig 1A). In total, RxR transitions would be possible, but because of the specific layout of the apartment, not all of these are observed. An observer watching the recorded sequence would see transitions of images (‘rooms’) 1 → 2 with probability $T_{2,1}^{*}$ or 1 → 3 with probability $T_{3,1}^{*}$, etc. The hidden ‘rule’ of sequence generation arises from the transition matrix $T_{i,j}^{*}$ (Fig 1B). However, at unknown moments in time the rule changes (with switch probability H, called ‘volatility’), akin to the switch from rule *A* → rule *B* in (1). Note that the set of images remains the same after a change point while the transition matrix changes [39, 47]. The above probabilistic task with rule switching is a generalization of established tasks in cognitive neuroscience of surprise [31, 84–87].

As opposed to an agent that selects actions to collect information, our observer is passively watching the image sequence. From the observed transitions between images, the observer learns which image (or images) to expect given the current one, i.e., estimate transition probabilities *T*_*i*,*j*_. This passive mode is ideal for a study of surprise because, in the context of neuroscience, it avoids any confounding factors arising from action selection [88] or reward [89] and, in the context of reinforcement learning theory, it avoids any complex interaction with models of curiosity, action selection policy or questions of model-based versus model-free reinforcement learning [90, 91]—simply because our observer does not choose actions. Once the set of possible transitions under a given rule has been learned, this knowledge could, of course, be used in model-based reinforcement learning, but this is not part of the tasks that we consider.

A typical sequence of rule switches is shown in Fig 1C where different rules *m* = 1, 2, 3, … correspond to different transition matrices $T_{i,j}^{*}\left(m\right)$. Inspired by experimental observations for passive learning in humans and animals [10, 24, 87, 92, 93], we assume that the (potentially unconscious) goal of observers is to predict possible next observations, i.e., estimate transition probabilities *T*_*i*,*j*_ that are as close as possible to the real probabilities $T_{i,j}^{*}\left(m\right)$. Our spiking neural network model (introduced in the next paragraph) implicitly encodes expectations about possible next stimuli in the set of synaptic weights. From this set of weights, we extract the expectations at time *t* in the form of a learned transition matrix *T*_*i*,*j*_(*t*) that can be compared to the currently active rule $T_{i,j}^{*}\left(m\left(t\right)\right)$ (Fig 1B). Input images are represented in the model by a simple code such that each image corresponds to a different subset of active sensory neurons. The expectations summarized in the learned transition matrix *T*_*i*,*j*_(*t*) are a prerequisite to extracting a surprise signal.

### A spiking network model

**Fig 2:**
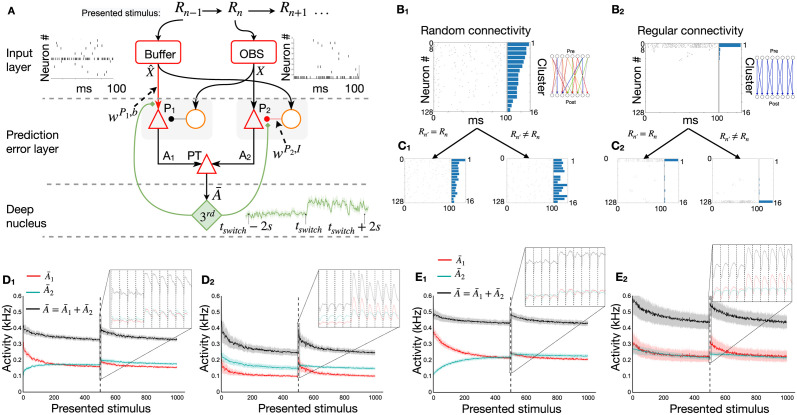
Neurons in prediction error layer respond to unexpected transitions. **A**. Spiking network model ‘SpikeSuM’. From top to bottom: Every 100ms stimuli change, giving rise to a sequence *R*_*n*−1_, *R*_*n*_, *R*_*n*+1_… The presently observed stimulus (*R*_*n*_, red box ‘OBS’) and the previous stimulus (*R*_*n*−1_, ‘Buffer’) are encoded with spike trains of 128 neurons each (16 sample spike trains shown). These spike trains are transmitted to two excitation-inhibition networks (prediction error layer) composed of pyramidal neurons (red triangles) and inhibitory neurons (orange circles). Pyramidal neurons in population *P*_1_ are excited (arrowheads) by the inputs representing the prediction $\hat{X}$ based on stimulus *R*_*n*−1_ and inhibited (round heads) by the current observation *X* whereas neurons in *P*_2_ are inhibited by the prediction $\hat{X}$ and excited by the current observation *X*. The activity *A*_1_ and *A*_2_ of populations *P*_1_ and *P*_2_ is transmitted to pyramidal tract neurons (PT), which low-pass filter the activity and transmit it to a group of neurons in a deep nucleus (green, labeled 3^*rd*^) which sends a neuromodulatory surprise signal back to the prediction error layer. Poorly predicted stimuli increase activity in the prediction error layer and indirectly accelerate, via the 3^*rd*^ factor, learning in the plastic connections (red lines). Inset: Time course of the 3^*rd*^ factor (green) over 4s before and after a rule switch at time *t*_*switch*_. **B**: Spike trains of all 128 pyramidal neurons in population *P*_2_ during a specific stimulus *R*_*n*_. The 128 neurons have first been ordered from highest to lowest firing rate and then clustered into groups of 8 neurons, with neurons 1 to 8 forming the first cluster. Right: Histogram of average firing rate per cluster (horizontal bars). **B_1_**: Random sparse connectivity from presynaptic neurons in the input layer to neurons in the prediction error layer. Inset: schematics, colors indicate connection strength from red (weak) to blue (strong). **B**_2_: Regular connectivity with binary connections. Inset: schematics, nonzero connections (blue) are organized in clusters of 8 neurons, but for readability, only 4 clusters of two neurons each are shown. **C**_1_ **and** **C**_2_: To compare the two networks, we show the spikes generated in response to a new stimulus *R*_*n*′_ while keeping the same order of neurons. For random connectivity (C1) spike plots are different if *R*_*n*′_ ≠ *R*_*n*_ but similar if *R*_*n*′_ = *R*_*n*_. The same holds for regular connectivity (C2). **D**_1_ **and** **D**_2_: Filtered activity of pyramidal neurons in populations *P*_1_ (red), *P*_2_ (cyan), and the total filtered activity $\bar{A}$ (black) as a function of time-averaged over 100 different sequences with a change point (switch of rule) after 500 presentation steps, for random (D1) or regular (D2) connectivity (parameter *K* = 2). Both networks indicate a surprising transition (dashed vertical line) by increased activity. Insets show the activity before and after the rule switch. **E**_1_ **and** **E**_2_: Same as in D_1_ and D_2_, but for the case of *K* = 4 possible next stimuli. Since predictions are less reliable, the activity $\bar{A}$ converges to higher levels.

The **Spike**-based **Su**rprise-**M**odulated (SpikeSuM_*rand*_) network model (Fig 2A) consists of an input layer with random projections onto excitatory and inhibitory neurons in a prediction error layer, and a deep nucleus (i.e., a cluster of neurons in the central nervous system located below cortex [68, 94]), e.g., the Locus Ceruleus [23], the ventral tegmental area [95] or higher-order thalamus [96, 97]. Neurons in the prediction error layer receive spikes from a first pool of *N* neurons encoding the currently observed stimulus and from another pool of *N* neurons in a memory buffer that encodes information on the previously observed stimulus (Material and methods). Synapses onto excitatory or inhibitory neurons have different weights. Two populations of pyramidal neurons *P*_1_ and *P*_2_, putatively located in cortical layers 2/3 [24], compare the weighted inputs of the current observation with the weighted inputs arising via connections from the memory buffer that we interpret as ‘predictions’. Population *P*_1_ is inhibited by the current observation and excited by the prediction coming from the buffer, whereas population *P*_2_ is excited by the current observation and inhibited by the prediction. Both populations project to a group of pyramidal tract (PT) neurons, putatively located in layer 5b [55, 98], which output a low-pass filtered version $\bar{A}$ of the summed neuronal activity. Since $\bar{A}$ reflects the combined outputs of populations *P*_1_ and *P*_2_, the output of PT neurons can be interpreted as a symmetric measure of ‘distance’ between prediction and observation (Material and methods). If a prediction is correct, excitation and inhibition balance each other so that the total activity $\bar{A}$ of all pyramidal neurons is close to zero.

In our model, the PT-neurons send the filtered network activity information $\bar{A}$ to an unspecified nucleus (Fig 2A) which sends back a neuromodulatory signal $3^{rd}\left(\bar{A}\right)$ that is broadcasted across the prediction error layer. We have checked that a large activity $\bar{A}$, caused by positive or negative prediction errors [21, 24, 92, 99, 100] indicates an unexpected transition. A transition is *unexpected* (‘surprising’) if the network has for example learned that after image ‘6’, the next possible images are 2,5,7 or 10 (Fig 1A), but the observed input corresponds to image ‘3’, indicating that a switch point has occurred. Indeed we find that the amplitude $3^{rd}\left(\bar{A}\left(t\right)\right)$ of the 3rd factor increases after a switch of rules (Fig 2A, inset). We, therefore, interpret $3^{rd}\left(\bar{A}\right)$ as a ‘surprise signal’. Note that the surprise signal is a function of activity in the prediction error layer—and therefore implicitly a function of the mismatch between excitation and inhibition.

To achieve E-I balance for *expected* transitions, we assume that activated *excitatory* synapses from the buffer (*b*) onto neurons in population *P*_1_ change according to an anti-Hebbian three-factor plasticity rule, modulated by the surprise signal,

$$\begin{array}{c} \Delta w^{P_{1},b}=-3^{rd}\left(\bar{A}\right)\;h^{\text{post}}\;{\bar{\text{EPSC}}}^{\text{pre}}, \end{array}$$ (2)

where ${\bar{\text{EPSC}}}^{\text{pre}}$ is the filtered sequence of (unsigned) excitatory postsynaptic currents (EPSCs) caused by the presynaptic spike train and *h*^post^ is the input potential of the postsynaptic neuron (for details, see Material and methods). Analogously, we assume that activated *inhibitory* (*I*) synapses onto neurons in population *P*_2_ change according to a Hebbian three-factor rule modulated by the surprise signal

$$\begin{array}{c} \Delta w^{P_{2},I}=3^{rd}\left(\bar{A}\right)\;h^{\text{post}}\;{\bar{\text{IPSC}}}^{\text{pre}}\; \end{array}$$ (3)

where ${\bar{\text{IPSC}}}^{\text{pre}}$ is the filtered sequence of (unsigned) inhibitory postsynaptic currents (IPSCs). For convergence properties of the two learning rules see Material and methods.

### A mismatch of excitation and inhibition yields an intrinsically generated surprise signal

Earlier theories have established that both Hebbian learning of inhibitory synapses [63] and anti-Hebbian learning of excitatory synapses [101] lead, for predictable inputs, to a stabilization of the firing rate of postsynaptic neurons at a low value. To check whether this holds also true for the above three-factor rules, we focus on a long stimulation sequence of 1000 presentation steps containing a single switch from rule 1 to rule 2 after 500 presentation steps. Consistent with earlier Hebbian theories, we observe that the SpikeSuM_*rand*_ network converges after about 450 presentation steps to a stationary state of low activity (Fig 2D1). Moreover, the switch between rules causes a sharp peak in the activity $\bar{A}$ (Fig 2D1, inset). Thus the activity $\bar{A}$ of PT-neurons can indeed be used to extract a surprise signal that is large for *unexpected* observations.

The predictability of the next stimulus is higher in a volatile sequence task with *K* = 2 possible transitions from a given observation (Fig 2D1) than in a task with *K* = 4 (Fig 2E1). Hence, the next stimulus becomes ‘more expected’, the prediction error is lower, and the population activity converges to a lower value in Fig 2D1 than in Fig 2E1; mean activity averaged over the last 100 presentation steps is 375Hz in Fig 2D1 versus 461Hz in Fig 2E1 (*p* < 10^−10^). This observation leads to experimentally testable predictions (S2 Fig).

We consider two different architectures for the connectivity from the input spike trains to the pools *P*_1_ and *P*_2_ of pyramidal neurons. The first one, SpikeSuM_*rand*_ (Fig 2B1 and 2C1), uses sparsely connected random projection weights from the input layer to the prediction error layer, whereas the second one (SpikeSuM Fig 2B2 and 2C2) has a simplified connectivity matrix with hand-wired binary weights implementing a direct representation of input stimuli by non-overlapping subsets of pyramidal neurons in the prediction error layer (See Material and methods). Despite the fact that activity is more localized in the network with the simplified connectivity, we find that the qualitative features of the population activity in the simpler network (Fig 2D2 and 2E2) are similar to those of the randomly connected network (Fig 2D1 and 2E1). In particular, the population activity increases for both connectivity patterns after a rule switch. Given the qualitative similarity of responses for the two connectivity patterns, we focus on the following on SpikeSuM with the simple regular connectivity as a reference because it is faster to simulate and easier to interpret.

### Activity in prediction error layer represents the present stimulus and predicted alternatives

**Fig 3:**
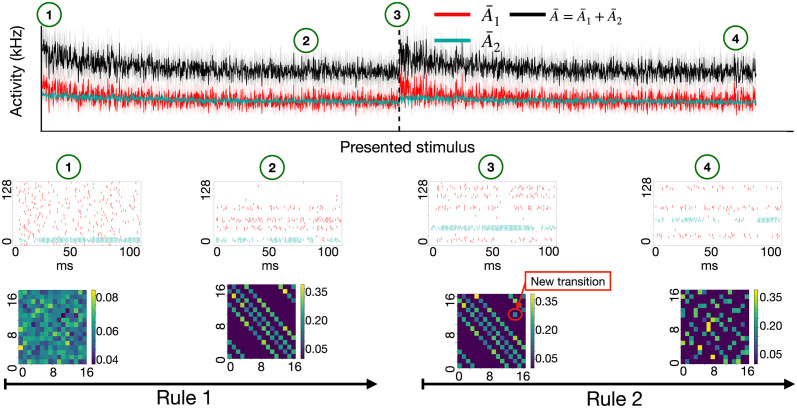
Neuronal responses depend on the present stimulus, the previous stimulus, and consistent alternatives to the present stimulus in a task with R=16 stimuli and *K* = 4 transitions possibilities and two rules. **Top**: Activity (arbitrary units) of populations *P*_1_ (green) and *P*_2_ (red) as well as the total activity $\bar{A}$ (black) of all pyramidal neurons. After 1500 presentation steps, the transition rule switches from rule 1 to rule 2. Each presentation step corresponds to the exposure to one stimulus for 100ms. **Middle**: Spike trains of pyramidal neurons during one presentation step, at different points during learning (from left to right): at the beginning (label 1) and end of the first episode with rule 1 (label 2) and beginning (label 3) and end of the first episode with rule 2 (label 4). If the observation is stronger than the prediction neurons in population *P*_2_ fire (blue dots); whereas if the observation is weaker than the prediction neurons in population *P*_1_ fire (red dots). Pyramidal neurons (16 per stimulus, 8 neurons each from *P*_1_ and *P*_2_) have been sorted according to stimulus numbers for visual clarity. **Bottom**. Matrix of transitions between stimuli decoded from the weights onto pyramidal neurons. At the end of the first presentation step after a change point (label 3), a new element (red arrow) has appeared in the transition matrix corresponding to the newly observed transition, *R*_*n*−1_ → *R*_*n*_. After some time with the novel rule, the new transition matrix is learned (label 4) and the old one is suppressed.

To illustrate the interpretation of the network with regular connectivity, we run the volatile sequence task of Fig 1A with R=16 different stimuli (*K* = 4) for 3000 presentation steps. In the beginning, the spike pattern across the populations *P*_1_ and *P*_2_ of pyramidal neurons in the prediction error layer looks noisy (Fig 3 middle left), but after a few hundred presentation steps with the first transition rule $T_{k,q}^{*}\left(1\right)$, the prediction error layer exhibits four active groups of neurons. These four groups represent the four possible transitions predicted from the *previous* stimulus, including the currently observed one (Fig 3 middle, second panel). Note that the predictions from the memory buffer of the previous stimulus excite neurons in population *P*_1_, whereas the current stimulus mainly excites neurons in population *P*_2_ (Fig 2A). Therefore, with *K* = 4 possible transitions, the currently observed stimulus is represented by a single group of neurons in population *P*_2_ whereas neurons in population *P*_1_ represent the three alternative predictions consistent with the previous stimulus. Thus, for the SpikeSuM network with regular connectivity, the activity in the prediction error layers reflects a column of the transition matrix $T_{k,q}^{*}$ where the fixed *q* denotes the previous stimulus (stored in the buffer) and the index *k* runs over the groups of neurons in the prediction error layer coding for stimulus *k*. Immediately after a switch to the new rule *m* = 2, a fifth cluster of active pyramidal neurons is observed. The five clusters correspond to the four wrong predictions (that have been learned with the previous rule and now cause negative prediction errors) and the currently observed (unexpected) stimulus under the new rule (which gives rise to a ‘positive prediction error’, in the sense that the current sensory input is stronger than the prediction [24]).

### Rapid learning after a rule switch

In order to decode the estimated transmission probabilities we use a decoding function that we construct as follows: First, we present each of the R stimuli, one at a time, for a long duration while blocking the output of the buffer population. The projections to population *P*_1_ in the prediction error layer cause an activity pattern $x^{P_{1}}$ across the pyramidal neurons in *P*_1_. We optimize a decoding matrix *D* such that $D^{P_{1}}x^{P_{1}}$ best approximates the 1-hot encoded stimulus number. Similarly, we determine a second decoding matrix *D*^*P*2^ to read out the activity from population *P*_2_. The two decoding matrices are kept fixed thereafter. In order to read out the *predicted* activity during the experiments, we block for a moment the inputs from the current observation so that neurons in *P*_1_ and *P*_2_ driven by the buffer and use the fixed decoding matrices $D^{P_{1}}$ and $D^{P_{2}}$. This yields the predicted stimulus labels that we use to construct the transition matrix in Fig 3 bottom. For mathematical details see ‘Materials and methods’. We note that, whenever the observation does not match the prediction, at least one of the populations *P*_1_ or *P*_2_ is active so that predictions are also visible in the spike patterns (Fig 3 middle).

A switch between rules causes a large activity, and turns on the neuromodulatory surprise signal $3^{rd}\left(\bar{A}\right)$ that leads to a fast update of the weights. We find that, after the rule switch, the new transition appears in the transition matrix already at the end of the first presentation step, i.e., after only 100ms (Fig 3 bottom, red circle in the graph). Thus, a single novel transition is sufficient to change the matrix (learning in ‘one shot’) by rapidly changing the synaptic weights (S2 Fig).

After spending some time with stimulus presentations under the new rule, the activity $\bar{A}$ of PT-neurons returns to a low value and the new transition matrix can be extracted from the weights onto pyramidal neurons in populations *P*_1_ and *P*_2_ (Fig 3, right, labeled 4).

### Modulation of plasticity by surprise supports rapid re-adaptation

**Fig 4:**
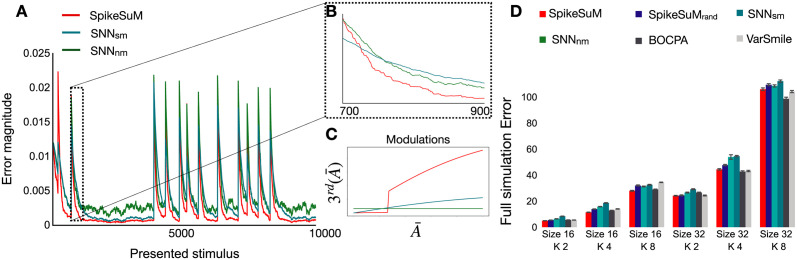
Rapid adaptation enabled by surprise-modulated three-factor plasticity. **A**: Error magnitude of the transition matrix (Frobenius norm between the true transition matrix T* and the estimated matrix *T*) as a function of time for the SpikeSuM model (red), and a Spiking Neural Network model (SNN) with the same architecture and number of neurons as SpikeSuM, but simple modulation (cyan SNN_*sm*_) or no modulation (green SNN_*nm*_), in a volatile sequence task with R=16 different stimuli and *K* = 4 possible transitions. Rule switches cause the occasional abrupt increases in error. The SpikeSuM network exhibits faster learning immediately after the switch as well as better convergence during periods when the rule stays fixed; volatility *H*=0.001. **B** Zoom on 200 presentation steps immediately after a rule switch. The red curve goes down faster and to a lower value than the other two. **C**: The surprise signal transmitted by the 3rd factor as a function of the activity $\bar{A}$ for three cases (red: SpikeSuM rule; cyan: simplified modulation rule; green: constant learning rate, no modulation). The parameters of all three rules have been optimized. **D** Average error over 10’000 presentation steps with volatility *H* = 0.001 for different values of R (size) and *K*. The performance of SpikeSuM is comparable to that of the Bayesian Online Change Point detection algorithm (BOCPA, black) and varSMile (grey) and better than SNN_*nm*_ or SNN_*sm*_. The results with random connectivity SpikeSuM_rand_ are shown in dark blue.

To understand whether the modulation of plasticity by surprise is necessary for the rapid re-adaptation after rule switches, we use a long sequence of 10,000 presentation steps to compare SpikeSuM with two simpler networks with the same architecture but different modulation (Fig 4A). In our reference model, the third factor $3^{rd}\left(\bar{A}\right)$ has two components that yield a slow modulation of plasticity for small $\bar{A}$ and a rapid one for large $\bar{A}$ (Fig 4C, red line). The two components enable rapid learning after an unexpected rule switch (Fig 4B), and slower, but sustained learning during a long phase with a fixed rule with a residual level of ‘expected uncertainty’ [33] caused by stochastic transitions compatible with the rule. In comparison, a network with an optimized, but constant learning rate (no modulation, SNN_*nm*_) converges after a switch of rules with a short delay (Fig 4A) towards a high-error solution. Moreover, a network with a simpler modulation SNN_*sm*_ shows fairly good convergence but adapts more slowly immediately after a switch (Fig 4B). We find that, within the family of tested functions, a 3^*rd*^ factor built of two components as in SpikeSuM is necessary to reach adaptation that is both fast and precise, but adding a third component does not further improve learning.

A systematic comparison shows that SpikeSuM and SpikeSuM_*rand*_ outperform SNN_*sm*_ and SNN_*nm*_ across various instantiations of the volatile sequence task (Fig 4D). Moreover, the performance of SpikeSuM is only slightly worse than that of the variational Bayesian algorithm varSMiLe [46] or the online Bayesian change point detection algorithm BOCPA [42] which are both surprise-based machine learning algorithms designed for near-optimal change-point detection (Material and methods). For the comparison with the above benchmark algorithms, the learning parameters of SpikeSuM and SpikeSuM_*rand*_ have been optimized separately for each paradigm. This may look unrealistic since in general the amount of stochasticity (characterized by the stochasticity parameter *K*) is not known upfront, or could even be different for different transitions within the same rule. We have checked that a network with fixed parameters can cope with a situation where stochasticity changes, within the same rule, from deterministic (*K* = 1) to stochastic (*K* = 2); see S2 Fig.

In summary, on the volatile sequence task without re-occurrence of the same rule, our spiking network with surprise-modulated learning shows faster relearning after a rule switch than the one without which suggests an essential role of surprise-modulation in rapid, yet precise, adaptation to changes in the stimulus statistics. Notably, the surprise signal is not some external variable but is extracted from the spiking activity of the network itself.

### Relation to behavioral surprise

**Fig 5:**
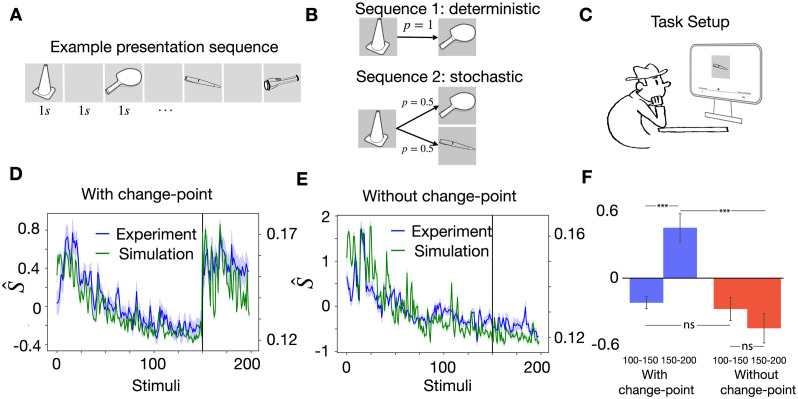
Behavioral surprise of human participants compared to simulated surprise. Example of an image sequence. Each image is presented for 1*s* followed by a 1*s* grey screen. Subjects are informed to focus on one specific image (e.g. ‘pen’) and the transition from there to the following image. **B** Sequence 1 is deterministic and used to familiarize the subject with the task. Sequence 2 has stochastic transitions so that each given image can be followed by one of *K* = 2 other images, with equal probability *p* = 0.5. **C** Participants observe the image sequence while attempting to predict the image following the pen and report their feeling of surprise continuously by moving a ‘Surprise slider’. Participants are randomly assigned to two different groups, with and without change points. **D** Scaled normalized surprise $\hat{S}$ reported by the 65 participants in group 1 (blue line: mean; shaded blue: variance) as a function of time (Methods), overlaid with appropriately scaled surprise in 60 simulations with SpikeSuM (green line: mean; shaded green: variance) using the same sequence as in the experiments with change point after 150 image presentations. **E** Same as D, but for the sequence **without** change-points. **F** Differences in the experimental data of participants are significant (t-test) in D between the 50 steps before and 50 steps after the change point (blue bars in F); not significant in E between the 50 steps before and 50 steps after step 150 in the absence of change point; and significant for the time steps 150–200 between D and E (blue vs. red bar in F). The symbol *** indicates *p* < 10^−5^, and ‘ns’ not significant.

In order to find out whether the surprise signal in our spiking network model is correlated with the subjective experience of surprise, we ran an experiment with 85 human participants viewing a sequence of images. Each of the images could be followed by one of *K* = 2 possible next images with probability *p* = 0.5 (Fig 5A and 5B). Participants were asked to focus on one image and report the feeling of surprise via a slider when seeing the following image (Fig 5C). The 25 participants who saw a sequence of 200 image presentations all generated with the same rule, reported (after an initial transient) a slowly decreasing surprise indicating that subsequent images were more and more expected. The 60 participants, however, who experienced a change of rule after 150 presentation steps, reported a strong increase in the subjective feeling of surprise. Importantly, the feeling of surprise reported by human participants (Fig 5D–5F) is strongly correlated with the simulated surprise signal in the SpikeSuM network if the model is stimulated with the same sequence (Pearson correlation 0.76 and 0.84 over all 200 time steps for experiments with or without change-point, respectively, calculated using several random samples of 20 participants). Thus the surprise signal in the SpikeSuM network links the notion of surprise in a behavioral experiment (Fig 5) with a functional role for modulating synaptic plasticity (Fig 4) at the level of neuronal circuits.

### Continual learning across rule switches is supported by the surprise signal

So far new rules involved *each time a new* transition matrix for the *same* set of stimuli. Each rule change induced overwriting of the previous transition matrix. We now explore how overwriting can be avoided. The first possibility is that different rules involve different stimuli. Suppose that the number of neurons in the input layer and in the prediction error layer is sufficient to accommodate 32 different stimuli, but rule A only uses 16 of these. If rule B uses 16 different stimuli, then a switch from rule A to rule B does not lead to overwriting. In this section, we consider a second scenario so as to study continual learning without overwriting.

**Fig 6:**
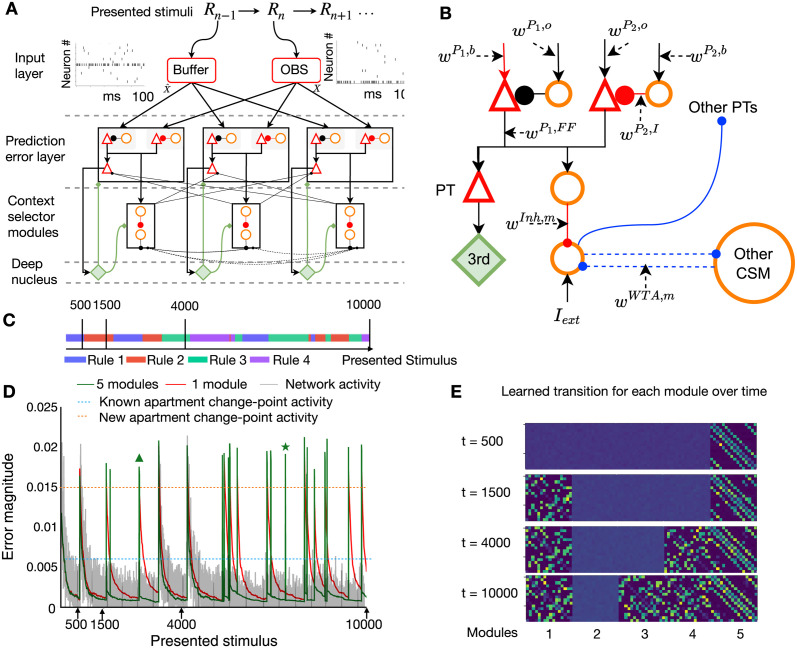
Continual learning across re-occurring rule switches. **A**: The SpikeSuM-C network is composed of four layers. The input layer receives the stimulus and connects to the prediction-error layer which is composed of several SpikeSuM modules (cf. Fig 2). A set of context selector modules (CSM) composed of dis-inhibitory networks is bidirectionally connected with the prediction-error layer. Each SpikeSuM module excites its corresponding CSM. A Winner-Take-All circuit in the CSM layer selects the least excited module. Inhibitory feedback weights from the CSM to the prediction-error layer inhibit the PT neurons of unselected SpikeSuM modules, but not the prediction-error neurons (see Material and methods). Red weights are plastic. Non-plastic weights are shown in black for feedforward, solid blue for feedback, and dashed blue for lateral inhibitory connections. **B**: Connectivity (schematic) within a single module. Disinhibition combined with WTA dynamics selects the module with the lowest activity in the prediction error layer. **C**: Sequence of rule switches as a function of time. **D**: Summed activity of all PT-cells (grey, arbitrary units) in a SpikeSuM-C network with 5 modules and error magnitude (green, mismatch between transition matrix in currently selected module and ground truth) during learning. When the second rule appears for the second time, the error exhibits a short spike (green triangle) indicating successful switching between modules. At rare moments (green star marks one of the examples) module switching is initiated at an inappropriate moment but stops immediately thereafter. The activity generated by the switch to an unknown rule is stronger (grey bars exceed the horizontal orange dashed line) than that of a previously observed one (grey bars barely reach the cyan dashed line). Red line: behavior of SpikeSuM (control, 1 single module). **E** Evolution of synaptic weight matrices over time for each of the five modules. After 500 time steps, the transition matrix of rule 1 has been stored in module 5, and transition matrices of other rules are added as they appear.

We now consider a task where all rules use the same stimuli (as before), but the same rules reappear *several* times. We study a meta-network composed of *M* SpikeSuM modules each acting as one of the rule memories (Fig 6A). We call this enlarged network SpikeSuM-C (for SpikeSuM with Context). Note that the set of stimuli (i.e., the different images) is the same for all rules so the context needs to be inferred from the observed sequences. Ideally, each module *m* ∈ *M* should focus on one of the contexts, i.e., a single transition matrix (rule). We postulate that in a well-functioning network, only predictions within the currently active rule are updated while multiple other contexts that were memorized before are left untouched and can be reused later when the same context reappears.

To implement this idea, we assume that a set of ‘context selector modules’ (CSMs) selects the specific module that should learn the observed transition (Fig 6B and Material and methods). The indirect coupling of context memories via the CSMs gives rise to a *Best-Predictor-Learns* (BPL) architecture, such that only the context module *m* with the *lowest* activity in the prediction-error layer updates its weights. Importantly, the prediction-error module with the lowest activity is the one with the best prediction for the currently observed transition. Moreover, the CSM have plastic weights that make a transition to a different module more likely if a surprising stimulus appears while the system is in a module for which it is ‘confident’ i.e., for which it has already encountered many stimuli.(Material and methods).

All CSMs compete with each other via standard Winner-Take-All dynamics [102], such that all CSMs are silent except one. However, none of the prediction error neurons is shut down by the competitive dynamics between CSMs, so an arbitrary population *p* in module *m* has a non-zero activity. To restrict synaptic plasticity to the prediction error module with the lowest activity, we hypothesize that the nucleus that broadcasts the third factor is organized in several segments, such that segment *m* sends a neuromodulatory signal $3^{rd}\left({\bar{A}}^{m}\right)$ to the corresponding prediction-error module *m*. Such a structure with localized feedback loops is compatible with the anatomy of the higher-order thalamus [68, 96, 97] or the ventral tegmental area [95]. More specifically, in our model the activity of populations *P*_1_ and *P*_2_ in the SpikeSuM module *m* excites segment *m* of the nucleus. In parallel, high activity of another CSM *m*′ ≠ *m* (i.e., *m*′ is the winner) inhibits PT neurons in the prediction-error layer of module *m* and hence suppresses segment *m* of the nucleus. But without neuromodulatory activity, plasticity does not occur in module *m*. Taken together, excitation and inhibition ensure that only the module *m*′ with the lowest prediction error updates its weights (Material and methods).

To illustrate the function of the network, we initialize it with 5 empty context modules and stimulate it with a stochastic sequence generated by switches between four different rules. Fig 6D shows that SpikeSuM-C learns the first rule as fast as SpikeSuM (equivalent to SpikeSuM-C with 1 module). Moreover, if a known rule reappears it re-activates an existing module instead of learning from scratch. Switches to a previously learned rule trigger a rapid switch of the network to the correct module. Finally, we find that if the number of learned rules is smaller than the number of allocated modules, empty modules stay untouched and therefore remain available for later use (Fig 6E).

The amplitude of the surprise signal after a switch to a previously encountered rule is smaller than that after a switch to a completely new rule (Fig 6D). In the first case, surprise leads to a switch to an existing module while in the second case to the recruitment of a previously untouched module. Thus, the surprise signals that are generated in the network are used by the same network to trigger learning or switching between context modules—all in an unsupervised manner (Material and methods for more details). We discuss in (Material and methods the time scale of switching. Additional tests with different values of the volatility *H* and stochasticity parameter *K* are summarized in Tables A and B in S1 Text. For the network to function well, it is important that there are at least as many context modules as potential rules (S1 Text).

### A modular network architecture avoids the stability-plasticity dilemma

Carpenter and Grossberg identified many years ago the stability-plasticity dilemma of brain plasticity: learning is either too slow to explain observed phenomena or, if fast, it leads to overwriting of earlier memories [77]. To solve the dilemma, Gershman et al. [35] have suggested a plasticity curve for memory formation that postulates memory modification for small prediction errors and memory protection for large prediction errors, leading to an inverted-U-shaped curve [35]. SpikeSum-C contains several populations of neurons, *P*_1_ and *P*_2_, that learn to respond to negative and positive prediction errors, respectively. Here we ask whether plasticity modulation by the third factor avoids the stability-plasticity dilemma in line with the hypothesis of Gershman.

**Fig 7:**
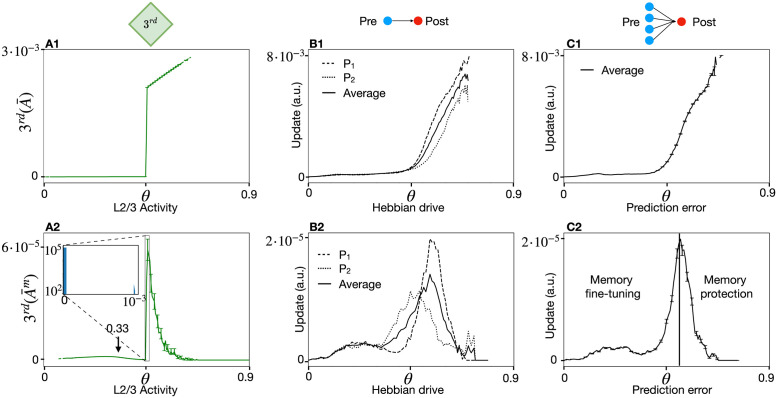
Synaptic plasticity as a function of prediction error has two regimes in SpikeSuM-C. **A1-A2**: The magnitude of modulation (3^*rd*^ factor) is shown as a function of the total activity $\bar{A}$ of layer-2/3 neurons for a SpikeSuM-C network with a single module (A1; equivalent to the original SpikeSuM) and for a SpikeSuM-C network with three modules (A2). The threshold *θ* is defined in Eq 14. Bars: standard error of the mean. The difference between the two curves (A1-A2) arises from the inhibition of model PT-neurons if they are not located in the winning module: in A1, the activity $\bar{A}$ of PT neurons always reflects the activity *A* of layer-2/3 neurons, in A2 it does not. Inset: Histogram of modulation amplitudes $3^{rd}\left({\bar{A}}^{m}\right)$ for values slightly above *θ*: the distribution of modulation amplitudes is bimodal with rare events of large modulation. Arrow: the peak is due to known transitions that remain after a rule change. **B1-B2**: The update magnitude |Δ*w*_*ik*_| of a specific synapse is shown as a function of the Hebbian drive $Re\text{tanh}\left(h_{i}\right)\cdot {\bar{\text{EPSC}}}_{k}$ i.e., the multiplication of postsynaptic membrane potential and the current influx caused by presynaptic spike arrival (long-dashed line, averaged over all neurons *i* in the postsynaptic population *P*_1_). Analogously, for postsynaptic population *P*_2_ (dotted line) and mean over both populations (solid line). **C1-C2**: The total amount of synaptic plasticity, represented by the update magnitude ∑_*k*_|Δ*w*_*ik*_| summed over all synapses onto an arbitrary neuron *i* is shown as a function of the prediction error, represented by the rectified and scaled membrane potential Retanh*h*_*i*_. In a network with a single module (C1), plasticity increases with prediction error so that large prediction errors after a context change lead to overwriting of existing memories. In the network with multiple modules (C2), the plasticity in the SpikeSum-C network exhibits two regimes: prediction errors between 0.1 and 0.4 generate small but non-negligible changes, and induce a refinement of existing memories, whereas for prediction errors above 0.6 existing memories are protected since other memories are created or changed. The error bars represent the 90% confidence interval of the mean. The vertical bar indicates the separation between the two regimes predicted by Gershman et al. [35].

We focus on synapses onto the layer 2/3 prediction error neurons in SpikeSuM-C and study the amplitude of the third factor as a function of the total activity in layer 2/3 (Fig 7A). In the original SpikeSuM with a single module, the third factor $3^{rd}\left(\bar{A}\right)$ increases monotonically once the total activity is larger than a threshold *θ* defined in Eq 14 (Fig 7A1). In SpikeSuM-C, however, the third factor jumps at *θ* to a large value and then decreases for higher values of activity (Fig 7A2). The reason is that, in the SpikeSuM-C network with multiple modules, a large activity of prediction error neurons in layer-2/3 of module *k* does not cause emission of neuromodulator in module *k* since a *different* module *k*′ ≠ *k* is the winner.

The third factor influences the amount of plasticity, but synaptic plasticity also requires the two Hebbian factors, i.e., ‘pre’ and ‘post’, to be non-zero; cf. Eqs (2) and (3). We define the Hebbian drive as the multiplication of ‘pre’ and ‘post’ where ‘pre’ represents the presynaptic activity ${\bar{\text{EPSC}}}^{\text{pre}}$ or ${\bar{\text{IPSC}}}^{\text{pre}}$ and ‘post’ the rectified and scaled postsynaptic membrane potential ${\left[\text{tanh}\left(h^{\text{post}}\right)\right]}_{+}$ where [.]_+_ is short-hand for rectification and tanh ensures scaling with a maximum of one. As a function of the Hebbian drive, the total amount of weight updates in populations *P*_1_ and *P*_2_ exhibits a monotonic increase in the SpikeSuM network with a *single* module, but a bell-shaped dependence in the SpikeSuM-C network with *multiple* modules (Fig 7B).

Finally, in order to relate our plasticity model to the hypothesis of Gershman [35], we ask whether we can find a similar bell-shaped curve as a function of the prediction error at the level of single postsynaptic neurons. We note that the prediction error is, by design of the network, represented by the membrane potential $h^{\text{post}}$ (where $h^{\text{post}}=0$ is the resting potential). Hence we study the update magnitude Δ*w* of all synapses onto a given postsynaptic neuron as a function of the rectified and scaled membrane potential ${\left[\text{tanh}\;h^{\text{post}}\right]}_{+}$ and average the result overall prediction error neurons (Fig 7C). Since the membrane potential encodes positive (in population *P*_2_) or negative (in population *P*_1_) prediction errors, the graph in Fig 7C can be interpreted as the total amount of synaptic plasticity (vertical axis) as a function of prediction error (horizontal axis). The small magnitude of synaptic changes for very large prediction errors (Fig 7C2) is functionally important because it leads to the protection of existing modules after a switch of context.

Therefore our model has translated a hypothesis at the cognitive level [35] into specific experimental predictions for synaptic plasticity at the circuit level. In an in-vivo experiment involving multiple contexts, presynaptic activation and postsynaptic membrane potential of putative prediction-error neurons should be monitored while the size of the synaptic connection is measured, e.g., by spine size estimation from optogenetic experiments. We speculate that in primary sensory areas, future experimental observations might resemble the qualitative features of SpikeSuM whereas in the frontal cortex or subcortical areas those of SpikeSuM-C.

## Discussion

Our network of spiking model neurons enables the rapid formation of context-dependent expectations in a paradigm of continual learning where rule switching occurs at unknown moments in time. Importantly, rapid adaptation becomes possible by surprise-modulated learning. In contrast to earlier implementations of surprise in cognitive neuroscience models [16, 31–33, 38, 40], surprise manifests itself in our spiking neural model by increased population activity caused by a momentary imbalance of excitation and inhibition [24, 63]. The surprise signal has two different roles in our model. First, it triggers the release of feedback signals (e.g., neuromodulators) that serve as ‘third factors’ in an unsupervised NeoHebbian learning rule [50, 51, 54]. Second, it initiates switches between modules and avoids overwriting old memories [35, 79, 80], since synaptic plasticity is dis-inhibited only in the module representing the current rule. We find that the protection of earlier memories of transition rules is possible only if the number of available network modules is larger than the number of different rules (S1 Text) Thus the number of modules limits the overall network capacity to encode different rules. If more rules than modules are encountered, all modules re-learn and adjust to the present rule, akin to catastrophic forgetting. If however, the number of rules is smaller to or equal to the number of modules, the modules focus on different rules so that earlier knowledge is not forgotten. Yet, each of the modules is not simply frozen, but remains plastic so as to enable further fine-tuning of ‘its’ rule. Each of the learned transition rules can be interpreted as a different context: given that the network is currently in, say, state number four, the most likely transition under the first rule (context one) might be to state seven, but under the second rule (context two) to state five. The network dynamics implicitly keeps a memory of the current context over short times that stabilizes learning while the surprise signal enables rapid switching if necessary.

In our approach, predictive coding is a consequence of our aim to extract a surprise signal from spiking activity—as opposed to classic approaches where predictive coding is a consequence of redundancy-reducing or energy-minimizing codes [64, 75]. Surprise requires expectations that arise from earlier experience. In our model, the sensory experience of the previous presentation step is represented in the buffer population while predictions are encoded in the connection weights. It is not necessary that the buffer population uses the same code as the observation population since the comparison of prediction and observation occurs via *plastic* synapses originating from the buffer. Our model does not specify whether the buffer population is located in the same area (e.g. cortical L5 cells [24]) or in some other area (e.g., prefrontal cortex [103, 104]). The anti-symmetric architecture of the prediction-error circuit in each module requires two separate excitatory and inhibitory pathways onto model neurons that extract positive and negative error prediction, similar to putative prediction error neurons in layer 2/3 of the sensory cortex [93, 105]. We propose that the activity of these neurons is summed, and potentially low-pass filtered, by layer 5b PT neurons [55, 98] which would then transmit the aggregated signal (‘surprise’) to other areas or nuclei that eventually trigger a feedback signal such as the release of a neuromodulator. While positive or negative prediction errors can be assigned to *single* neurons, surprise in our model is extracted from the *aggregated* unsigned prediction error—available by a summation over large groups of neurons.

Our model is a conceptual one and makes no specific predictions on the type or origin of these feedback signals. However, candidate sources for such feedback signals could be acetylcholinergic neurons in nuclei of the basal forebrain and brain stem, potentially linked to arousal and plasticity [68, 94, 106]; noradrenergic neurons in Locus Ceruleus linked to cognition, attention, network reorganization, and gating of plasticity [23, 107, 108]; serotonergic neurons in the Raphe nuclei linked to surprise [14]; dopaminergic neurons in the ventral tegmental area linked to reward [109]; or populations of neurons in the higher-order thalamus potentially linked to consciousness or predictive processing [68, 96, 97]. At least for the last two it is known that the population is not homogeneous but structured [95, 97] which is a necessary condition for the proposed model of switching between different rules encoded in different modules. Even though dopamine is largely correlated with reward and reward prediction error [89, 109], dopamine has also been linked to novelty and potentially surprise [109, 110]. On the other hand, dopamine can also be triggered by activity in Locus Ceruleus [111, 112], a nucleus that is traditionally associated with noradrenaline [107]. Hence, a one-to-one mapping between neuromodulators and functional roles should not be expected [52].

Predictions in our model are encoded at two levels, i.e., in the weights of synaptic connections and the activity pattern of excitatory neurons in the prediction-error layer (Fig 3). While the model was not designed to reproduce experimental data of frontal cortex neurons, several aspects of the activity patterns in the SpikeSuM-C model are qualitatively consistent with delay activity [104], implicit encoding of associations [103], and mixed activity profiles [113] which enables to decode from the population activity the current rule, the present input, the previous stimulus, and alternative observations consistent with the previous stimulus but inconsistent with the present input. A limitation of our current implementation of the model is the assumption of a buffer population that keeps the memory of the previous event *R*_*t*−1_. The weights onto pyramidal neurons in the prediction layer implicitly estimate the transition matrix $\hat{P}\left(R_{t}|R_{t-1}\right)$. The combination of discrete representation time steps with an explicit buffer population has enabled us to extract the transition matrix by a local learning rule modulated by a third factor. It is conceivable that the buffer population could be replaced by a recurrent network where information about the past reverberates and is available from the current network state *R*_*t*−1_ ≈ *F*(*state*_*t*_) where *F* is a decoding function. In such a scenario, the expectation about the current state would have to be encoded by a modified transition matrix $\tilde{P}\left(R_{t}|F\left(state_{t}\right)\right)$. Whether a standard three-factor rule is sufficient in this case, or whether a bio-plausible learning rule that approximates backpropagation through time [59, 114] is preferable, needs further research.

A further limitation is the organization of the model circuits in an anti-symmetric fashion. While positive and negative prediction errors need to be processed by separate circuits [24], the circuitry in Fig 2A has several biologically implausible features. First, the inhibitory neurons in the model circuit implement exact sign inversion. This restriction could be relaxed in a randomly coupled recurrent network where inhibitory neurons connect to each other to implement a *K*-winner-take-all circuit. Second, plasticity is restricted to a peculiar subset of connections. This condition could be relaxed as shown by the following thought experiment: We assume a large number of neurons in the input and prediction error layer and suppose that connections (YES or NO) with plasticity (ON or OFF) are assigned randomly to all eight connection types, i.e., buffer to excitatory *P*_1_, buffer to excitatory *P*_2_, buffer to inhibitory neurons projecting to *P*_1_, buffer to inhibitory neurons projecting to *P*_2_, and analogously four connections types from the current observation to *P*_1_ and *P*_2_. Then a small, but non-negligible fraction of all connections would have the ‘correct’ combination as shown in Fig 2 in a sea of many other connections. Thus, only a small fraction of neurons in the prediction error layer would actually encode positive or negative prediction errors, consistent with experimental data in layer 2/3 [115]. The other connection types in such a random connectivity scheme are likely to specialize to other tasks. While the other connection types may increase the noise in the surprise signal, there is no reason to believe that they would systematically cancel the surprise signal established by the ‘correct’ combination of connections. We therefore assume that the activity of neurons embedded in other connection patterns ‘average out’ and do not contribute to the surprise signal that marks change points. Whether this assumption is justified, or whether additional feedback processes are needed to further select the ‘correct’ wiring patterns remains an open question.

Another limitation is that, in particular for SpikeSum-C, parameters depend on the level *K* of stochasticity of the rule. While SpikeSum-C with a fixed set of parameters is able to cope with stimuli that combine deterministic (*K* = 1) with probabilistic transitions with *K* = 2 (S2 Fig), the switching between modules is no longer reliable if parameters optimized for stochasticity *K* = 4 are used for stimuli with *K* = 2 or vice versa. Interestingly, for our behavioral experiments with human participants we anectodically observed that paradigms that combine *K* = 1 and *K* = 2 work well (Fig 5) whereas paradigms with *K* > 2 do not. This suggests that parameters of brain circuit that extract transition rules might by default be tuned to low stochasticity. Whether, and how, parameters can be automatically adjusted to rules with large, but variable, levels of stochasticity is an open research question. One suggestion is that slow variables with low-pass filter characteristics keep track of the variance of the transition probabilities and feed the variance signal back to adjust hyperparameters.

A distinction between expected and unexpected uncertainty has been proposed in the literature on reward-based learning [33, 116]. Analogously, we can define expected and unexpected uncertainty in the absence of rewards. In our volatile sequence task, the expected uncertainty depends on the number *K* of possible next stimuli whereas the unexpected uncertainty corresponds to unpredictable switches between rules. For *K* = 1, the expected uncertainty vanishes. For *K* > 1, the level of expected uncertainty is, after learning, represented in our model by the remaining activity of excitatory neurons in the prediction error layer which could be tested in experiments (S2 Fig). Expected uncertainty can also be visible as a non-zero tonic level of the surprise signal (i.e., the 3rd factor). The unexpected uncertainty is represented by sharp peaks in the activity of the prediction error neurons (Fig 6D).

Detecting unpredictable switches in the rules governing the momentary environment is a challenge for both artificial neural networks [76] and biological brains [14, 104]. If rule switching is not detected, for example, because of reduced serotonergic signaling, behavior exhibits reduced adaptation speed [14] or even obsessive-compulsive signatures [14, 117]. Surprise in our model is putatively related to mismatch negativity in EEG signals. Interestingly, schizophrenia patients exhibit a reduced mismatch negativity [118] and a reduced capacity to make valid prediction [119–121]. In our model, missing surprise signals lead to an impairment of memory formation and consolidation, potentially linked to deficits in schizophrenia patients [122–125].

Definitions of surprise in a probabilistic framework [4] have previously been used to explain adaptation to rule switching [42–45]. However, these definitions cannot be directly applied to spiking neural networks since a correct normalization of probability distributions is difficult to maintain within spiking networks [126, 127] and the calculation of a distance, or Kullback-Leiber divergence, between two probability distributions [4, 128] is even harder. Surprise-driven neural networks for adaptive decision-making [129] or neural particle filters for adaptive perception [130] are not easily extendable to networks of spiking neurons. Our approach extracts from the activity of spiking neurons a qualitative surprise signal that can be interpreted as a measure of observation-mismatch surprise [4] without a direct link to probability distributions. In summary, surprise, i.e., a response of the brain to a stimulus that occurs against expectations [1–4], is a phenomenon of relevance similar to that of reward. Similar to reward and reward expectations [89], surprise must be detected by neuronal networks in the brain and transformed into modulatory signals that influence synaptic plasticity. Our conceptual model study shows how surprise detection and modulation of plasticity can be implemented in spiking neural networks and how these networks can be used for memory formation, memory protection, and prediction of upcoming inputs, in the absence of reward.

## Material and methods

### Two volatile sequence tasks

In the volatile sequence tasks (Fig 1A), a sequence of stimuli is generated by a doubly stochastic Markov chain. At each presentation step, a stimulus with index *q* is chosen from a finite set of R different inputs, 1≤q≤R. Given a stimulus *q* at presentation time step *n*, a stimulus *k* at presentation step *n* + 1 is chosen with probability $T_{k,q}^{*}$ where T* is the transition matrix that summarizes a given rule. At each presentation step, rules switch stochastically with probability *H* ≪ 1, called the volatility of the rule. We often refer to the moment of rule switch as a ‘change point’. From the point of view of the observer, switches are unexpected and potentially cause a high surprise.

While the theory is more general, we often visualize stimuli as static wallpaper images collected by a video camera that is moved randomly across an apartment composed of R rooms (Fig 1A), each enabling *K* possible transitions to other rooms. Rooms have distinct wallpapers. The stimulus *R*_*n*_ stands for the wallpaper in the room seen at presentation step *n*. The transitions are stochastic and follow:

$$\begin{array}{c} P\left(R_{n+1}|R_{n}\right)=\{\begin{array}{c} \frac{1}{K}\;\text{if}\;\text{stimuli}\;R_{n+1}\;\text{and}\;R_{n}\;\text{refer}\;\text{to}\;\text{connected}\;\text{rooms}\\ 0\;\text{otherwise,} \end{array} \end{array}$$ (4)

We assume periodic boundary conditions, e.g., room 4 in Fig 1A is a neighbor to rooms 1,3,8 and 16. Thus the layout of the apartment defines the hidden rule of allowed transitions between stimuli. In particular, a transition matrix generated from a given apartment has the property that for each starting stimulus *q*, the elements $T_{k,q}^{*}$ either vanish or take a value $T_{k,q}^{*}=1/K$ with constraints $\sum_{k}T_{k,q}^{*}=1$ and $T_{q,q}^{*}=0$. In the theory below we do not assume that the transition matrix is symmetric, even though whenever we simulate an apartment with a two-dimensional layout and *K* = 4 (or a 1-dimensional apartment with *K* = 2), then the matrix is symmetric $T_{k,q}^{*}=T_{q,k}^{*}$.

We design two tasks with different switching patterns. In both tasks, the number of different stimuli is fixed and equal to R. For the first task (‘volatile sequence task without re-occurrence of rules’), at each change point, all stimulus numbers are randomly shuffled. Thus at each change point, a new transition rule is generated while keeping the number *K* of possible next stimuli fixed (visualized as a new layout of the apartment in Fig 1). For the second task (‘volatile sequence task with re-occurrence of rules’), we first randomly shuffle the set of R stimulus numbers *M* times (i.e., we first create *M* different apartments that all use the same wallpapers). This procedure gives rise to *M* different transition rules. At each change point, we randomly choose one of the *M* − 1 possible other rules. Thus, the number of potential transition rules is finite. The first task implies that having a memory of a past rule is vain as there is a very low probability of encountering the same rule multiple times. Hence, an adaptive algorithm with rapid forgetting is suited to solve this task. For the second task, a suitable algorithm should memorize context-dependent predictions and quickly re-activate the correct context after each rule switch.

In the simulations in the main text, we use symmetric transition matrices with neighborhood relations that can be visualized as apartments with either R=16 or R=32 rooms and vary the number *K* of allowed transitions per room between *K* = 2 and *K* = 8. The terms ‘apartment’, ‘room’, and ‘wallpaper’ are for illustration purposes only since each stimulus is represented in the model by a unique neuronal input pattern (see below).

### Spike trains of sensory neurons

To simulate the volatile sequence task with R discrete stimuli (‘wallpapers of rooms’), we translate the stimuli into spiking patterns of abstract ‘sensory’ neurons: each stimulus is represented by a distinct cluster of *m* = 8 neurons with an elevated firing rate of 100Hz. (We may think of these ‘sensory’ neurons as the output of a multi-layer network with wallpaper images as input and 8-hot coding as output, but we do not implement such a preprocessing network.) Each stimulus presentation lasts for 100ms (=1 presentation step), and thereafter a new input stimulus is presented to the network. The network input layer is composed of two populations of ‘sensory’ neurons: a population of observation neurons and a population of buffer neurons (see Fig 2). Both populations consist of m×R Poisson neurons. Note that we use *m* = 8 neurons per cluster to have a good estimation of the firing rate; however, for a network of rate neurons, it would be sufficient to use a single neuron per stimulus (1-hot coding).

In a network of 8×R presynaptic neurons per sensory population, the first cluster of 8 neurons represents the first stimulus (*q* = 1) of the volatile sequence task, the second cluster consists of neurons 9 to 16 the second one and so forth. For each observation, neurons in one of the clusters will spike with firing probability 0.1 at each time step of *dt* = 1ms (firing rate 100Hz), whereas all other neurons fire with probability *ϵ* ≪ 0.1 at each time step. Note that, sensory neurons in the buffer population have the same behavior as those in the observation population except that active neurons encode the stimulus number of the previous observation.

### Transmission from sensory neurons to prediction error neurons

Each spike *z*_*k*_ in neuron *k* of one of the sensory populations triggers an unsigned square EPSC of length *l* = 4ms which is transmitted to neurons in the prediction error layer consisting of two populations *p* ∈ {*P*_1_, *P*_2_}. The total input current *I*_*i*_ into neuron *i* of the prediction error layer is

$$\begin{array}{c} I_{i}^{P_{1}}={\hat{x}}_{i}^{P_{1}}-x_{i}^{P_{1}}, \end{array}$$ (5)

if neuron *i* is in population P_1_ and

$$\begin{array}{c} I_{i}^{P_{2}}=x_{i}^{P_{2}}-{\hat{x}}_{i}^{P_{2}}, \end{array}$$ (6)

if neuron *i* is in population P_2_. Here and in the following the variable $x_{i}^{p}$ without hat refers to the observed, and the one with hat to the predicted input current to neuron *i* and *p* ∈ {*P*_1_, *P*_2_} refers to the two populations in the prediction error layer. Specifically,

$$\begin{array}{c} x_{i}^{p}\left(t\right)=\sum_{k}w_{ik}^{p,o}{\text{EPSC}}_{k}^{o}\left(t\right), \end{array}$$ (7)

is the input from sensory neurons in the observation population to neuron *i* in population *p* of the prediction error layer where ${\text{EPSC}}_{k}^{o}\left(t\right)$ is 1 if neuron *k* in the observation population has fired in the last 4ms and $w_{ik}^{p,o}$ are fixed observation weights. Similarly,

$$\begin{array}{c} {\hat{x}}_{i}^{p}\left(t\right)=\sum_{k}w_{ik}^{p,b}{\text{EPSC}}_{k}^{b}\left(t\right). \end{array}$$ (8)

is the input from sensory neurons in the buffer population to neuron *i* in population *p* of the prediction error layer where ${\text{EPSC}}_{k}^{b}\left(t\right)$ is 1 if neuron *k* in the buffer population has fired in the last 4ms and $w_{ik}^{p,b}$ are plastic weights driven by the plasticity rule of Eq 19. We refer to ${\hat{x}}_{i}^{p}\left(t\right)$ as the (learned) prediction and to $x_{i}^{p}\left(t\right)$ as the (representation of the present) observation. To simplify the notations we drop in the following the time argument *t* and replace

$$\begin{array}{c} x_{i}^{p}≜x_{i}^{p}\left(t\right)\\ {\hat{x}}_{i}^{p}≜{\hat{x}}_{i}^{p}\left(t\right) \end{array}$$ (9)

### Spiking neuron model

**Fig 8:**
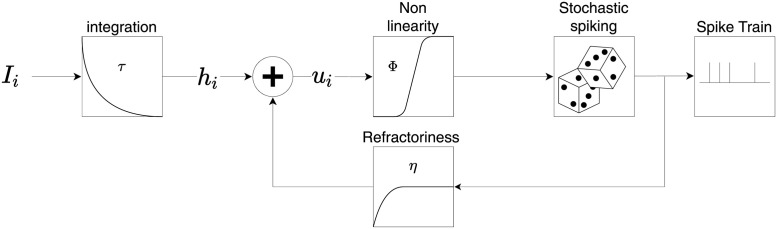
Spike Response Model of neurons in the prediction error layer. Each postsynaptic neuron receives an input current *I*_*i*_. This current is integrated, with membrane time constant *τ*, to obtain the input potential *h*_*i*_. The actual membrane potential of the neuron *u*_*i*_ is the combination of both the input potential and a refractory function *η*, where *η* is a strong negative potential activated after a spike, forcing the neuron to stay silent for a while. The spike times are then randomly drawn with probability *ϕ*(*u*_*i*_) generating the spike train of neuron *i*.

Neurons in the prediction error layer are described by the Spike Response Model SRM_0_ [131, 132]. Each prediction error neuron *i* receives an input current $I_{i}^{p}$ where *p* stands for *P*_1_ or *P*_2_; cf. Eqs 5 and 6. The input current is then integrated to obtain the input potential (Fig 8)

$$\begin{array}{c} \tau \frac{dh_{i}^{p}}{dt}=-h_{i}^{p}+I_{i}^{p}\;⟶\;h_{i}^{p}\left(t\right)=\frac{1}{\tau }\int_{-\infty }^{t}e^{\frac{s-t}{\tau }}I_{i}^{p}\left(s\right)\text{ds} \end{array}$$ (10)

Combining the input potential with a refractory kernel *η* leads to the membrane potential

$$\begin{array}{c} u_{i}^{p}\left(t\right)=\eta \left(t-t_{i}^{\prime }\right)+h_{i}^{p}\left(t\right), \end{array}$$ (11)

where $t_{i}^{\prime }$ stands for the *last* firing time of post-synaptic neuron *i*, and $\eta \left(t-t_{i}^{\prime }\right)=-e^{-\frac{\left(t-t_{i}^{\prime }\right)}{\tau }}$ is an exponential refractory function, preventing the neuron to fire again right after a spike. Spikes are generated stochastically with probability

$$\begin{array}{c} P\left(z_{i}=1\right)=\phi \left(u_{i}\right)={\left[\text{tanh}\left(u_{i}\right)\right]}_{+} \end{array}$$ (12)

per time steps of *dt* = 1*ms* where *ϕ* is the activation function of the neurons and [*x*]_+_ = *x* for *x* > 0 and zero otherwise. Eqs (10), (11) and (12) define the Spike Response Model of the prediction error neurons.

### Two connectivity patterns onto the prediction error layer: Random and regular

The first projection pattern (SpikeSuM_*rand*_) is sparse random connectivity (with density 0.1) and weights uniformly drawn between 0 and 1. In other words, for each of the 256 postsynaptic neurons in the prediction error layer, we draw an input connection to a specific presynaptic neuron with a probability of 10 percent and then connect the two neurons with a random weight (Fig 2). Since in our standard simulations, we have 16 different stimuli and each stimulus is represented by a distinct cluster of *m* = 8 presynaptic neurons, the average number of input connections to a neuron in the prediction error layer is 0.1·m·R=12.8 with a mean weight of 0.5. Thus, in the prediction error layer stimuli are represented by overlapping groups of neurons of different firing rates (coarse coding).

The second projection pattern is a regularly structured connectivity pattern (SpikeSuM). Presynaptic neurons are, as before, separated in R clusters of *m* = 8 neurons each, but each cluster projects (with binary weights) to a different group of 8 neurons in the prediction error layer. In other words, both pre-and postsynaptic layers are composed of 8R neurons such that different stimuli are represented in the prediction error layer by distinct, non-overlapping groups of neurons (Fig 2).

### SpikeSuM network architecture

Eqs (5) and (6) show that $I_{i}^{p}=0$ if and only if ${\hat{x}}_{i}^{p}=x_{i}^{p}$, for *p* ∈ {*P*_1_, *P*_2_}. Note that ${\hat{x}}_{i}^{p}$ is the prediction arising from the activity of the buffer population whereas $x_{i}^{p}$ is the present observation. Hence, the total input is minimal if the prediction coincides with the observation. A wrong prediction increases the activity in at least one of the two populations in the prediction error layer: if ${\hat{x}}_{i}^{p}>x_{i}^{p}$ for many neurons in *p* = *P*_1_, then many neurons in population *P*_1_ have a positive input current and nonzero spiking activity; on the other hand, if ${\hat{x}}_{i}^{p}<x_{i}^{p}$ for many neurons in *p* = *P*_2_, then many neurons in population *P*_2_ have a positive input current and nonzero spiking activity. Because of the rectification at the transition from neuronal input $I_{i}^{p}$ to output spikes (Eq 12), the two populations *P*_1_ and *P*_2_ complement each other. A natural way to estimate the overall prediction error of the network is therefore to collect the spikes of both populations *P*_1_ and *P*_2_. We assume that the population of PT-neurons acts as a linear filter and transmits a mean activity $\bar{A}$ defined as

$$\begin{array}{cc} \tau_{\bar{A}}\frac{d\bar{A}}{dt} & =-\bar{A}+c\sum_{p=P_{1},P_{2}}\sum_{i}{\text{EPSC}}_{i}^{p}, \end{array}$$ (13)

where ${\text{EPSC}}_{i}^{p}$ denotes the square excitatory postsynaptic current of neuron *i* of population *p* and $\tau_{\bar{A}}$ and *c* are constants. In our model, $\bar{A}$ provides the total drive of neurons in a deep nucleus that receives dense input connections from PT cells. The neurons in the deep nucleus send back a broadcast signal that measures the total surprise

$$\begin{array}{c} \prime \text{surprise}\prime =\eta_{1}\text{tanh}\left(\bar{A}\right)+\eta_{2}\text{tanh}\left(\bar{A}\right)\Theta \left(\bar{A}-\theta \right)=3^{rd}\left(\bar{A}\right) \end{array}$$ (14)

where $3^{rd}\left(\bar{A}\right)$ is a nonlinearly increasing function of $\bar{A}$, Θ is the Heaviside step function and *η*_1_, *η*_2_, *θ* are fixed hyper-parameters. Since the surprise signal modulates learning, we refer to it as a 3^*rd*^ factor that gates plasticity in NeoHebbian three-factor learning rules [54].

The third factor, composed of two non-linear components, could either be interpreted as a single neuromodulator with a complex nonlinearity or alternatively as the combined action of two neuromodulators involved in surprise-based learning [33]. Following the terminology of [33], the adaptation to the *expected* uncertainty (e.g., stochastic transitions to one of the possible next stimuli under a fixed rule) could be controlled by the action of acetylcholine [described in our model by the term $\eta_{1}\text{tanh}\left(\bar{A}\right)$], whereas the adaptation speed to the *unexpected* uncertainty (i.e. a rule switch) could be controlled by the action of norepinephrine [turned on in our model if $\bar{A}>\theta$].

### SpikeSuM learning rule: Derivation of Hebbian factors

We aim for a NeoHebbian plasticity rule with three factors [54], i.e., a rule that combines traces of pre-and postsynaptic activity with a modulation of the learning rate. As indicated above, a good prediction of the present observation is indicated by the small current in Eqs (5) and (6) or, similarly, by a small value of the input potential $h_{i}^{p}$ of all pyramidal neurons in populations *P*_1_ and *P*_2_; cf. Eq (10). We therefore minimize the loss function

$$\begin{array}{c} L=\int_{t^{start}}^{t^{stop}}\hat{L}\left(t\right)\;dt=\int_{t^{start}}^{t^{stop}}\frac{1}{2}\sum_{p=P_{1},P_{2}}\sum_{i}{\left[h_{i}^{p}\left(t\right)\right]}^{2}\;dt \end{array}$$ (15)

where *t* is time and runs from the beginning *t*^*start*^ to the end *t*^*stop*^ of the experiment. Optimization is implemented as online gradient descent with respect to the weights $w_{ik}^{p,b}$ that project from neuron *k* in the buffer population to neuron *i* in population *p* of the prediction error network. We recall that weights $w_{ik}^{p,o}$ from observation neuron *k* to neuron *i* are fixed. We present here all the calculations for *p* = *P*_1_ only. For the population *P*_2_ one just needs to add a minus sign. The integral over time corresponds to a batch rule; for stochastic gradient descent (online rule) we can focus on an arbitrary point in time and apply the chain rule of differentiation

$$\begin{array}{c} \frac{\partial \hat{L}\left(t\right)}{\partial w_{ik}^{P_{1},b}}=h_{i}\left(t\right)\frac{\partial h_{i}^{P_{1}}\left(t\right)}{\partial w_{ik}^{P_{1},b}} \end{array}$$ (16)

where we can evaluate the derivative using Eqs (5) and (8)

$$\begin{array}{c} \frac{\partial h_{i}^{P_{1}}\left(t\right)}{\partial w_{ik}^{P_{1},b}}=\int_{-\infty }^{t}e^{\frac{s-t}{\tau }}\frac{\partial I_{i}^{P_{1}}\left(s\right)}{\partial w_{ik}^{P_{1},b}}ds\underset{Eq.(8)}{=}\int_{-\infty }^{t}e^{\frac{s-t}{\tau }}{\text{EPSC}}_{k}^{b}\left(s\right)ds \end{array}$$ (17)

Since EPSCs have a rectangular shape with duration *l* we can evaluate further

$$\begin{array}{c} \frac{\partial h_{i}^{P_{1}}\left(t\right)}{\partial w_{ik}^{P_{1},b}}=\sum_{t_{k}^{\prime }\leq t}e^{\frac{-t}{\tau }}\int_{t_{k}^{\prime }}^{t_{k}^{\prime }+\Delta_{k}}e^{\frac{s}{\tau }}ds=\sum_{t_{k}^{\prime }\leq t}e^{\frac{-(t-t_{k}^{\prime })}{\tau }}\left[e^{\frac{\Delta_{k}}{\tau }}-1\right]≜{\bar{\text{EPSC}}}_{k}^{b}, \end{array}$$ (18)

where ${\bar{\text{EPSC}}}_{k}^{b}$ is a low-pass filtered version of the EPSC, $t_{k}^{\prime }$ are the spike times of neuron *k* and $\Delta_{k}=min\left(t-t_{k}^{\prime },l\right)$. We now apply online gradient descent with an update amplitude proportional to the variable 3^*rd*^ (‘learning rate’) and the step size *dt*

$$\begin{array}{c} \frac{dw_{ik}^{P_{1},b}}{dt}=-3^{rd}\;h_{i}^{P_{1}}\;{\bar{\text{EPSC}}}_{k}^{b}\;\;\text{in}\;\text{population}\;P_{1},\\ \frac{dw_{ik}^{P_{2},I}}{dt}=\;\;+3^{rd}\;h_{i}^{P_{2}}\;{\bar{\text{IPSC}}}_{k}^{b}\;\;\;\text{in}\;\text{population}\;P_{2}. \end{array}$$ (19)

The above NeoHebbian rule combines a trace of the incoming ${\text{EPSC}}_{k}^{b}$ (presynaptic factor) with the momentary input potential $h_{i}^{P_{1}}$ (rather than the spike time) of the postsynaptic neuron (postsynaptic factor): these are the two Hebbian factors. Repeating the same derivation for the plastic inhibitory connections in population *P*_2_ leads to the second update rule in Eq (19) which is analogous to the first one except for the sign. In standard stochastic gradient descent, the learning rate 3^*rd*^ could be fixed or slowly decrease over time as learning proceeds (‘freezing’), and also depend (via a momentum term) on the recent history. However, in our model, the learning rate increases whenever the prediction fails (indicated by a large prediction error) so we refer to the learning rate 3^*rd*^ as a surprise-driven neuromodulator. To summarize, we have a three-factor learning rule with the following properties: (i) ${\text{EPSC}}_{k}^{b}$ (respectively ${\text{IPSC}}_{k}^{b}$) limits the weight update to active connections; (ii) *h*_*i*_ is the local signed prediction error and goes to zero if the prediction for neuron *i* is correct; (iii) finally, 3^*rd*^ is a function of the global unsigned prediction error which is sent back as ‘surprise’ to the full network; see main text and section (Mathematical Details below).

### SpikeSuM learning rule: Third factor

There is no fundamental reason that a learning rate should be fixed as long as each update step (in the batch-rule) decreases the loss [133]. However, in an online gradient descent rule, we have to make sure that all observations get an appropriate statistical weight during the update. In particular, we have to ensure that none of the observations is systematically ignored. This could happen if the learning rate 3^*rd*^ vanished whenever a specific stimulus appears. Such a problem is not a hypothetical one, because of the rectification of the neuronal gain function; cf. Eq 12. Suppose that for a given stimulus, the observation $x_{i}^{P_{1}}$ is larger than the prediction ${\hat{x}}_{i}^{P_{1}}$ for all neurons in population *P*_1_. In this case, none of the neurons in population *P*_1_ would respond. If we were to use a third factor that is proportional to the activity *A*_1_ of population *P*_1_ (e.g., if we set 3^*rd*^ = *βA*_1_), then this stimulus would never lead to an update.

However, the dependence of the third factor $3^{rd}\left(\bar{A}\right)$ upon the total population activity $\bar{A}$ of the prediction error layer together with the anti-symmetric architecture avoids this problem. Whenever the observation does not match the prediction, at least one of the populations, either *P*_1_ or *P*_2_, will be turned on. This is true throughout the simulation because (i) there are many plastic weights that code for each stimulus (e.g., with regular connectivity and R=16 different stimuli, we have 64 weights coding for each stimulus in each of the two populations); (ii) all synaptic weights in both populations are initialized in the range [0, 1]; (iii) the update rule Eq (19) is symmetric for both populations (i.e., if the excitatory weights onto a neuron in *P*_1_ increase, then the inhibitory weights onto a neuron in *P*_2_ decrease) which ensures that the symmetries at initialization remain throughout learning.

Thus whenever predictions and observations do not match, the total activity $\bar{A}$ conveys a prediction error signal which leads to a non-zero learning rate $3^{rd}\left(\bar{A}\right)$ that is identical for *all* weights.

### Benchmark algorithms

We compare the performance of our network to several state-of-the-art algorithms (Fig 4). For fairness of comparison, each of these algorithms uses surprise-based online adaptation to detect change points induced by rule switching. BOCPA [42] is a Bayesian online algorithm for exact inference of the most recent change point. It is a message-passing algorithm that infers the probability distribution over the run time since the last switch. It is known to be optimal on average for long simulations. VarSMiLe [46] is a variational approximation [134] of BOCPA that uses the Bayes Factor surprise S_*BF*_ [46] to detect change points. VarSMiLe does not need message-passing (as implemented in BOCPA) and has a closed-form update rule similar to the SMiLe rule [39].

We also compare SpikeSuM with networks of the same architecture but with a simplified function for the third factor. The function 3^*rd*^, introduced in Eq (3), scales the amount of plasticity. The first line in the following equation gives the definition while the other lines the simplifications considered

$$\begin{array}{ccc} 3^{rd}\left(\bar{A}\right) & =\eta_{1}\text{tanh}\left(\bar{A}\right)\Theta \left(\bar{A}\right)+\eta_{2}\text{tanh}\left(\bar{A}\right)\Theta \left(\bar{A}-\theta \right), & \text{for}\;\text{SpikeSuM}\\ 3^{rd}\left(\bar{A}\right) & =\eta_{1}\text{tanh}\left(\bar{A}\right), & \text{for}\;{\text{SNN}}_{sm}\\ 3^{rd}\left(\bar{A}\right) & =\eta_{1} & \text{for}\;{\text{SNN}}_{nm}\;. \end{array}$$ (20)

This comparison of 3^*rd*^ factors allows us to investigate the impact of modulation on the learning. The differences in the number of networks parameters can be found in Table 1.

**Table 1:** Networks parameters for simulations with R stimuli.

	Presynaptic neurons	Postsynaptic neurons	3^*rd*^ hyper-parameters
SpikeSuM	8×R	128	3
SpikeSuM_*rand*_	8×R	256	3
SNN_*sm*_	8×R	128	1
SNN_*nm*_	8×R	128	1

### Simulation parameters and comparison of algorithms

Simulations are obtained by running networks composed of, 8×R, presynaptic neurons (so that 8 neurons have sustained spiking for each stimulus), and 128 postsynaptic neurons (256 for random connectivity). The presynaptic neurons have a firing rate of 100Hz if representing the observed stimulus and the squared EPSCs (IPSCs for inhibitory neurons) last for 4ms. The integration time of the input potential *τ* = 10*ms*. See Table 2.

**Table 2:** Summary of fixed network parameters.

Parameters list	value
refractoriness *η*	0.9
presentation *T*_*pres*_	100
input integration *τ*	10
input rate *ν*	0.1
length E(I)PSC *l*	4

Hyper-parameters (*η*_1_, *η*_2_, *θ*) of SpikeSum as well those for VarSMiLe and BOCPA have been optimized using the python library *scikit-optimize* [135] minimizing $\sum_{n}\left|\right|T_{n}-{\hat{T}}_{n}{\left|\right|}_{F}^{2}$, where *T*_*n*_ is the transition matrix defining the rule at time step *t* and ${\hat{T}}_{n}$ the estimated transition matrix extracted from the weights and ||.||_*F*_ the Frobenius norm.

The full SpikeSuM network with modulation by the standard third factor has more parameters than the network with no modulation (nm), or simple modulation (sm) as indicated in Table 1.

### Behavioral experiments

*Experimental setup*: Participants sit in front of a screen and observe a sequence of images on the screen. Images are black-and-white drawings of objects of daily life on a grey background from the Bank of Standardized Stimuli (BOSS) [136]. Each image is presented for 1*s* followed by a 1*s* grey screen. Participants are informed to focus on one specific image (e.g. ‘shoe’) and the transition from there to the following image (Fig 5A–5C). Participants are informed to predict the image following the shoe and asked to report their feeling of surprise continuously throughout the task by moving a ‘Surprise slider’ horizontally.

*Familiarization task*. Sequence 1 is deterministic, i.e., given image *n* only one image (*K* = 1) is possible as subsequent input (Fig 5B). All participants first perform the familiarization task once before turning to the experimental task.

*Experimental task*. Sequence 2 has stochastic transitions so that each given image can be followed by one of *K* = 2 other images, with equal probability *p* = 0.5. Sequence 2 can either contain a change of rule after 150 image presentations (group 1, with change point) or not (group 2, control). 60 participants were randomly assigned to group 1 and 25 participants to group 2. Data was collected on the platform ‘prolific’, courtesy of Michael Herzog and Wei-Hsiang Lin, EPFL, according to the Ethics rule of the EPFL ethics committee.

*Normalized subjective surprise $\hat{S}$*. All participants in a given group see the same realization of the stochastic sequence. In the first processing step, the reported raw surprise *S* is, for each participant, normalized to zero mean and unit variance during time steps 1 to 150 to yield a normalized subjective surprise

$$\hat{S}\left(t\right)=\frac{S\left(t\right)-E[{\text{S}}_{\left[1:150\right]}]}{\sigma [S_{\left[1:150\right]}]}$$

where *S*(*t*) is the raw surprise on the slider at time step *t*. In the second step, we calculate the average over all participants in a given group.

### Context modules: Architecture of SpikeSuM-C

SpikeSuM-C is an extension of the original SpikeSuM network and is composed of *M* SpikeSuM modules (Fig 6A).

**Fig 9:**
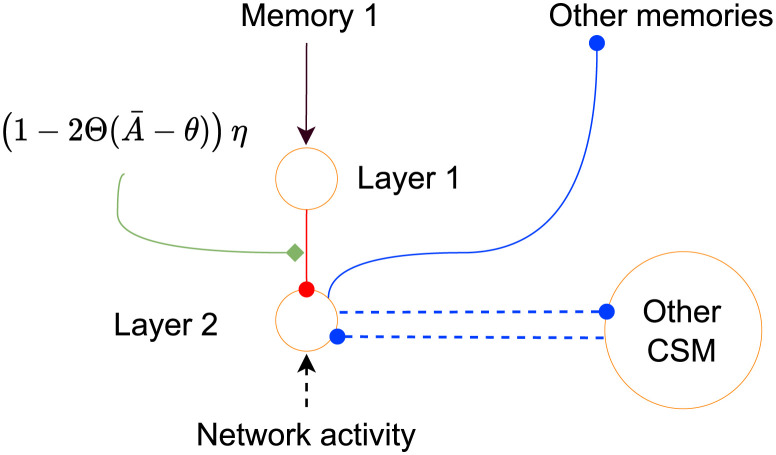
Context selector module (CSM). Each CSM contains two layers of inhibitory neurons. Layer 1 receives excitatory input from the corresponding SpikeSuM module. Layer 2 receives inhibition from layer 1 and lateral inhibition from layer 2 of other CSMs. The more excitation a CSM receives, the lower the activity in layer 2. Because of WTA dynamics implemented by lateral inhibition, the CSM module with the lowest excitation is selected, inhibits other CSMs, and shuts down the plasticity of other SpikeSuM modules. The red weights are plastic and can be interpreted as a ‘commitment’ to the selected module. The network activity represents the activity across all SpikeSuM modules and supports the WTA dynamics.

Each context selection module (CSM) has two layers, schematically shown in Fig 9. Information flows from the prediction error layer of module *m* into layer L1 of the CSM with the same index *m*. The output from layer L2 of the CSM with index *m* inhibits other CSMs with index *m*′ ≠ *m* and also inhibits the PT neurons of other modules *m*′ ≠ *m*. All $N_{\text{CSM}}$ spiking neurons in each of the two layers of one CSM are described by the Spike Response Model in Eq 10. We now discuss the different components in turn.

*Inputs to the CSM)*. The first layer (L1) of CSM *m* receives excitatory input from prediction error module *m* via feedforward synapses $W_{i,k}^{p,m,FF}$ (Fig 6B) that connect neuron *k* in population *p* ∈ {*P*_1_, *P*_2_} to neuron *i* in layer L1,

$$\begin{array}{c} I_{\text{L1},i}^{m}=a_{1}[\sum_{p}\sum_{k}W_{ik}^{p,m,FF}\;{\text{EPSC}}_{k}^{p,m}-\beta ] \end{array}$$ (21)

where *β* > 0 is a parameter. Neurons in layer L1 of module *m* send inhibitory input to the second layer (L2) of the same module via weights $W_{ik}^{Inh,m}$. But layer L2 in module *m* also receives inhibitory input from layer L2 of other modules *m*′ ≠ *m*. The inputs to neurons in layers L1 and L2 are

$$\begin{array}{c} I_{\text{L2},i}^{m}=a_{2}[-a_{3}\sum_{k}W_{ik}^{Inh,m}{\text{IPSC}}_{\text{L1},k}^{m}-a_{4}\sum_{m^{\prime }\neq m}\sum_{k}W_{ik}^{WTA,m^{\prime }}{\text{IPSC}}_{\text{L2},k}^{m^{\prime }}+a_{5}I_{\text{ext}}]. \end{array}$$ (22)

where *a*_1_, *a*_2_, *a*_3_, *a*_4_, and *a*_5_ are fixed positive parameters and ${\text{IPSC}}_{\text{L2},k}^{m^{\prime }}$ denotes the amplitude of the IPSC from neuron *k* in layer L2 of module *m*′ to neuron *i* in layer L2 of module *m*. The negative sign of inhibition has been made explicit as a minus sign in front of *a*_3_ and *a*_4_, Mutual inhibition implements a Winner-Takes-All (WTA) circuit where the least inhibited module stays selected whereas the other ones are silenced. $I_{\text{ext}}=\sum_{p}\sum_{m}\sum_{k}\;{\text{EPSC}}_{k}^{p,m}$ is the sum over all possible spikes in the prediction error populations (across all context modules) and serves as a positive bias that triggers the WTA dynamics.

*Outputs of the CSM*. Neurons *k* in CSM *m*′ sends inhibitory weights of magnitude $W_{ik}^{WTA,m^{\prime }}$ to L2-neurons other CSMs; see Eq (22) above. Moreover, the L2-neurons of the CSM with index *m*′ send also inhibitory input of fixed amplitude *J* to PT neurons in module *m*:

$$\begin{array}{c} \tau_{{\bar{A}}^{m}}\frac{d{\bar{A}}^{m}}{dt}=-{\bar{A}}^{m}+c\sum_{p=P_{1}^{m},P_{2}^{m}}\sum_{i}{\text{EPSC}}_{i}^{p,m}-J\sum_{m^{\prime }\neq m}\sum_{k}{\text{IPSC}}_{L2,k}^{m^{\prime }}, \end{array}$$ (23)

The sums run over all neurons *j* of all CSM other than the one with index *m*. We chose J = 20 for strong inhibition. We recall that the activity of PT-neurons ${\bar{A}}^{m}$ directly influences the third factor $3^{rd}\left({\bar{A}}^{m}\right)$ and hence modulates plasticity in module *m*.

*Synaptic plasticity*. The neurons in SpikeSuM module *m* are updated following

$$\begin{array}{c} \Delta w_{ik}^{P1,b,m}=-3^{rd}\left({\bar{A}}^{m}\right)\;h_{i}^{m}\;{\bar{\text{EPSC}}}_{k}^{P_{1},m}\;\text{in}\;\text{population}\;P_{1},\\ \Delta w_{ik}^{P2,I,m}=3^{rd}\left({\bar{A}}^{m}\right)\;h_{i}^{m}\;{\bar{\text{IPSC}}}_{k}^{P_{2},m}\;\;\;\;\text{in}\;\text{population}\;P_{2}. \end{array}$$ (24)

for each SpikeSuM module. The function form of 3^*rd*^(.) is the same as in Eq (14). Suppose for the moment that *m* is the active module. As a result of the strong inhibition from other modules (cf. Eq (23)), the third factor $3^{rd}\left({\bar{A}}^{m}\right)$ is positive for module *m* whereas $3^{rd}\left({\bar{A}}^{m^{\prime }}\right)=0$ for modules *m*′ ≠ *m*. Thus, the third-factor limits plasticity to the winning module. The selection of the winning module is done in the CSM network (Fig 9) via the WTA dynamics. The net result is a Best-Prediction-Learns (BPL) dynamics. It is a combination of dis-inhibitory feedforward connectivity within a module and lateral Winner-Take-ALL (WTA) dynamics between modules.

While the weights *W*^*p*,*m*,*FF*^ and $W^{WTA,m^{\prime }}$ are fixed at a value of one, the connections $W_{ik}^{Inh,m}$ from L1 to L2 are plastic. The inhibitory connections $W_{ik}^{Inh,m}$ are potentiated by a Hebbian rule modulated by a third factor and depressed by an unspecific decay term with decay rate *α*

$$\begin{array}{c} \Delta W_{ik}^{Inh,m}=\eta \;g^{3^{rd}}\left({\bar{A}}^{m}\right)\;{\text{IPSC}}_{L2,i}^{m}\;{\text{IPSC}}_{L1,k}^{m}-\alpha \left(W_{ik}^{Inh,m}-W_{0}^{Inh}\right). \end{array}$$ (25)

where $g^{3^{rd}}\left({\bar{A}}^{m}\right)$ is a third factor modulating plasticity (Fig 9). We call the weights $W_{ik}^{Inh,m}$ the confidence weights. Indeed, the product ${\text{IPSC}}_{L2,i}^{m}{\text{IPSC}}_{L1,k}^{m}>0$ drives the potentiation as long as neurons in both L1 and L2 are active; i.e. when the module *m* is selected. During this phase, we consider that the module builds its confidence about being a ‘good predictor’ for the current rule. After learning a rule for some time, $W_{ik}^{Inh,m}$ is much larger than its initial weight $W_{0}^{Inh}$. The term $\alpha (W_{ik}^{Inh,m}-W_{0}^{Inh})$, *α* ≪ 1, implements weight decay on a long timescale so that a module that is not used for a long time is slowly forgotten.

The function $g^{3^{rd}}\left({\bar{A}}^{m}\right)$ in Eq 25 allows to influence the direction of learning. We choose

$$\begin{array}{c} g^{3^{rd}}\left({\bar{A}}^{m}\right)=(1-2\Theta ({\bar{A}}^{m}-\theta )) \end{array}$$ (26)

*Network function*. The least active SpikeSuM module (i.e., the one yielding the best prediction) is chosen by the CSM network as the module that learns. Here, *a*_3_ governs the overall strength of the feedforward dis-inhibitory drive from the SpikeSuM module. The connection strength *a*_4_ controls the strength of lateral inhibition in the WTA circuit. The common bias *I*_ext_ accounts for the variability in the network activity so that the WTA dynamics works equally for predictable and unpredictable inputs and is not hindered by random variations due to spiking noise. The parameter *a*_2_ is a scaling parameter that we found useful in setting up the simulations. The choice of parameters is discussed below in **SpikeSuM-C parameters**.

Suppose that module *m* has been selected for some time, but now suddenly a higher prediction error in SpikeSuM module *m* occurs. This causes an increase of activity in L1 and leads to strong inhibition (because the weights had been potentiated earlier) of neurons in L2 so that the WTA mechanism rapidly ‘un-selects’ this module in favor of another one. Note that postsynaptic neurons in L2 of a CSM that lost the WTA competition are silenced so that connections to these neurons are no longer potentiated. The net result of the plasticity rule is that modules that have never been chosen in the past have connection weights that are still close to their initial values—and these modules can then be later selected by the WTA dynamics for new tasks.

### Analysis of switch point dynamics

We may ask ourselves how the network detects outliers that trigger a switch of modules. To study this, let us focus on the presentation step of 100ms during which the first outlier occurs, and analyze the Eq 22 in a rate model with constant input. We assume WTA dynamics. Let *Z* be the maximum activity of the PT neurons of the currently active module during the observation of an outlier and Z^*L*1^ the one in the first layer of the context detector. The dynamic in layer 2 of the active module is then

$$\begin{array}{c} \tau_{L_{2}}\dot{h^{L_{2}}}=-h^{L_{2}}+a_{2}\left[-a_{3}Z^{L_{1}}+a_{5}I_{ext}\right]. \end{array}$$ (27)

We assume that *a*_5_ is small enough so that $a_{2}\left[-a_{3}Z^{L_{1}}+a_{5}I_{ext}\right]<0$. Then over time neurons in layer 2 of this module will be silenced as soon as $h^{L_{2}}$ passes below zero, which implies that the observation triggers a switch-point. Hence the parameter *a*_2_ together with the time scale $\tau_{L_{2}}$ determine the resilience of the model to outliers. Indeed, the smaller *a*_2_ and the longer $\tau_{L_{2}}$, the more observations are required to detect a change point. For small $\tau_{L_{2}}$ and large *a*_2_, an outlier will be detected in a single presentation step and will lead to immediate switching of modules. In S1 Text we show the success rate of SpikeSuM-C as a function of the parameter *a*_2_ in a paradigm with *K* = 2 potential next states, but with transitions that are biased rather than balanced between the two possible next states.

So far we looked at a strong outlier that indicates a switch. At the other extreme would be a slow drift. For example, while each of the R inputs is encoded by *m* Poisson neurons (e.g, *m* = 8) after some time the code shifts so that 1 of the *m* neurons that have coded for stimulus *R*_*n*_ has changed identity and now codes for stimulus *k*(*n*) where *k*(*n*) is some permutation of stimulus indices. After several of such minor code switches effectively a different transition rule is implemented even though the rule would be represented by the same module.

### SpikeSuM-C parameters

Simulation results reported in Figs 6 and 7 are obtained in a paradigm with volatility *H* = 0.001 by running networks with the parameters summarised in Tables 3 and 4. The presynaptic neurons have a firing rate of 100Hz if representing the observed stimulus and the squared EPSCs and IPSCs last for 4ms. The integration time of the input potential *τ* = 10ms. The code is available on Git Hub (https://github.com/martinbarry59/SpikeSuMNet).

**Table 3:** Summary of SpikeSuM-C parameters with stochasticity parameter *K* = 2. The volatility parameter *H* = 1/1000 is used in the main text (middle column). Further results with *H* = 1/500 and *H* = 1/2000 can be found in Table A of S1 Text.

Context selection module parameters	H = 1/500	H = 1/1000	H = 1/2000
*a*_1_	0.256	0.22	0.22
*a*_2_	0.05	0.05	0.05
*a*_3_	4	4	4
*a*_4_, *a*_5_	20	20	20
*α*	1e-06	1e-06	1e-06
*β*	0.045	0.045	0.045
*η*	0.0003	0.00025	0.0003
$W_{max}^{Inh,m}$	0.11	0.08	0.09
SpikeSuM parameters			
*η*_1_	1e-05	1e-05	1e-05
*η*_2_	0.005	0.005	0.005
*θ*	0.45	0.45	0.45
*W*_*init*_	60	60	60
c	40	40	40
EI-neurons	128	128	128

**Table 4:** Summary of SpikeSuM-C parameters with stochasticity parameter *K* = 4. The volatility parameter *H* = 1/1000 is used in the main text (middle column). Further results with *H* = 1/500 and *H* = 1/2000 can be found in Table B of S1 Text.

Context selection module parameters	H = 1/500	H = 1/1000	H = 1/2000
*a*_1_	0.07	0.07	0.07
*a*_2_	0.05	0.05	0.05
*a*_3_	4	4	4
*a*_4_, *a*_5_	20	20	20
*α*	1e-06	1e-06	1e-06
*β*	0.055	0.055	0.055
*η*	0.0008	0.0007	0.0006
$W_{max}^{Inh,m}$	0.1	0.11	0.13
SpikeSuM parameters			
*η*_1_	1e-06	1e-06	1e-06
*η*_2_	0.002	0.002	0.002
*θ*	0.5	0.5	0.5
*W*_*init*_	9	9	9
c	40	40	40
EI-neurons	512	512	512

## Mathematical details: Decoding and transition probabilities

We claim that the anti-symmetric architecture of the prediction error layer in SpikeSuM together with the three-factor learning rule makes the weights converge to a solution that reflects the main features of the hidden rule defined by the transition matrix $T_{k,q}^{*}$ between stimuli. As before we consider a doubly stochastic process where the transition rules change with a small probability *H* per presentation step and each transition rule is defined by the transition matrix $T_{k,q}^{*}$. The amount of stochasticity of a given rule is controlled by a parameter *K*. For example,*K* = 4 means that four possible next stimuli can follow a specific stimulus *q*. We want to show that, for a transition matrix $T_{k,q}^{*}$ with *K* entries of value 1/*K* per column and zero entries otherwise, the weights onto the pyramidal neurons in the prediction error layer are adjusted such that all possible transitions are predicted proportional to their statistical probabilities. We will also show how to decode the predictions of the network.

### Preliminaries: Encoding of stimuli and decoding of predicted transitions

As an abstract encoding of stimuli, we use 1-hot encoding. If the total number of stimuli is R, then a specific stimulus *q* (with 1≤q≤R) is encoded by an R-dimensional vector $R_{q}\in {\left\{0,1\right\}}^{R}$ with the *q*th component equal to 1 and all other components equal to zero. The transition matrix $T^{*}\in R\times R$ that describes the probability of a transitions from stimulus **R**_*q*_ to stimulus **R**_*k*_ has elements $T_{k,q}^{*}$ defined as

$$\begin{array}{c} T_{k,q}^{*}=\text{Prob}\left(R_{k}|R_{q}\right)\; \end{array}$$ (28)

with $\sum_{k}T_{k,q}^{*}=1$ for all *q*. The set of stimuli $\left\{R_{1},...,R_{R}\right\}$ represented by 1-hot coding vectors defines an orthogonal basis in an R-dimensional vector space which gives rise to the following properties of the transition matrix T*. First, multiplication of the matrix with the stimulus vectors from both sides gives back the transition

$$\begin{array}{c} R_{k}T^{*}R_{q}=T_{k,q}^{*} \end{array}$$ (29)

and, second, one-sided multiplication with a stimulus **R**_*q*_ gives a vector **R**_.|*q*_ with non-zero elements for all those stimuli that can follow **R**_*q*_

$$\begin{array}{c} R_{.|q}=T^{*}R_{q}=\sum_{k}R_{k}T_{k,q}^{*}\;. \end{array}$$ (30)

We interpret **R**_.|*q*_ as the code of ‘consistent next stimuli’ that can follow stimulus *q*. It represents the *q*th column of the transition matrix T* and can be expressed as a linear sum over the one-hot-coded stimuli **R**_*k*_. In particular, for *K* = 4, the vector on the left-hand-side of Eq (30) contains four non-zero entries (with a value of 1/4 each) that represent the four possible stimuli after the stimulus with index *q*.

The actual encoding of stimuli in the input layer of the SpikeSuM network corresponds to m-hot encoding, since stimulus *R*_*q*_ is represented in the input layer by a cluster of *m* neurons that fire at a high rate (*ν*=100Hz); cf. **Spike trains of sensory neurons**. For the sake of simplicity of the arguments below, we assume that the neurons representing other stimuli *R*_*k*_ ≠ *R*_*q*_ are inactive (*ϵ* → 0) when stimulus *R*_*q*_ is observed. Thus we can think of the input representation of stimulus *q* as an *m*-hot encoding $V_{q}=P^{1\to m}R_{q}\in {\left\{0,1\right\}}^{mR}$, where *P*^1→*m*^ is the rectangular expansion matrix from 1-hot encoding to m-hot encoding transforming the R-dimensional space of stimuli into a mR-dimensional space of input neurons.

We now turn to the representation of stimuli in the prediction error layer. For the SpikeSuM network with *regular* connectivity, the representation in the prediction error layer is also an *m*-hot encoding in each of the two populations *P*_1_ and *P*_2_. However, to keep our arguments general we will also include the case of *random* connectivity. From Eq (7) we know that the input neurons in the observation population drive neuron *i* in population *p* of the prediction error layer with a current $x_{i}^{p}\left(t\right)=\sum_{k}w_{ik}^{p,o}{\text{EPSC}}_{k}^{o}\left(t\right)$. We collect the set of neurons *i* in population *p* into a vector **x**^*p*^, and the weights $w_{ik}^{p,o}$ into a matrix *W*^*p*,*o*^ and write the vector equation

$$\begin{array}{c} x^{p}\left(t\right)=W^{p,o}{\text{EPSC}}^{o}\left(t\right)\;. \end{array}$$ (31)

Let us consider a time point *t*_*n*_ located close to the end of the *n*th presentation step. Furthermore, let us suppose that during the *n*th presentation step stimulus $R_{q(t_{n})}$ was observed. Here *q*(*t*_*n*_) denotes the index of the stimulus in presentation step *n*.

We exploit the *m*-hot encoding to write for the mean activity pattern in population *p*

$$\begin{array}{c} E_{S}\left[x^{p}\left(t_{n}\right)\right]=W^{p,o}P^{1\to m}R_{q(t_{n})}\;\nu \;l \end{array}$$ (32)

where $E_{S}\left[x\right]$ denotes the expectation over stochastic spiking of the Poisson neurons in the input layer,*W*^*p*,*o*^ is the matrix of fixed connectivity weights to the pyramidal neurons in the prediction error layer, *ν* is the firing rate of the active neurons, and *l* is the duration of the rectangular EPSC. Similarly, the expected prediction generated by connections from neurons in the buffer population to those in population *p* ∈ {*P*_1_, *P*_2_} is

$$\begin{array}{c} E_{S}\left[{\hat{x}}^{p}\left(t_{n}\right)\right]=W^{p,b}P^{1\to m}R_{k(t_{n-1})}\;\nu \;l. \end{array}$$ (33)

Since we would like to interpret activity patterns in terms of the stimuli, we introduce hypothetical decoding weights *D*^*p*^ from the space of neuronal activities (in one of the pyramidal populations in the prediction error layer, *p* ∈ {*P*_1_, *P*_2_}) to the space of stimulus labels in 1-hot coding. We choose decoding weights such that encoding followed by decoding forms an auto-encoder for arbitrary stimuli *R*_*q*_:

$$\begin{array}{c} R_{q}=D^{p}\;W^{p,o}P^{1\to m}R_{q} \end{array}$$ (34)

With these decoding weights fixed, the read-out with the matrix *D*^*p*^ enables us to interpret the momentary activity **x**^*p*^(*t*_*n*_) of neurons in the prediction error layer in terms of stimulus labels; to see this compare the right-hand side of Eq (33) with Eq (34). Note that the decoding weights are an interpretation tool, but not implemented in the network (even though it would be easy to learn them, for example with the perceptron learning rule).

In order to interpret the *predicted* activity $E_{S}\left[{\hat{x}}^{p}\left(t_{n}\right)\right]$ in terms of stimulus labels, we use the *same* decoding weights *D* as for the observed activity

$$\begin{array}{c} {\hat{R}}_{.|k(t_{n-1})}=D^{p}\underset{E_{S}\left[{\hat{x}}^{p}\left(t_{n}\right)\right]/\left(\nu \;l\right)}{\underset{︸}{W^{p,b}P^{1\to m}R_{k(t_{n-1})}}}, \end{array}$$ (35)

where *k*(*t*_*n*−1_) is the index of the stimulus during presentation step *n* − 1 and ${\hat{R}}_{.|k(t_{n-1})}$ is the prediction of stimuli in step *n*, given the stimulus with index *k*(*t*_*n*−1_) in step *n* − 1. These predicted stimulus labels enable not only the decoding of predictions in the figures of the Results section but are also at the core of the following theory insight.

### Weights after convergence reflect transition probabilities

Loosely speaking, we claim that given that the stimulus in the previous time step *t*_*n*−1_ was **R**_*q*_, the predictive input from the buffer population can be decoded and represents the average of the possible next stimuli consistent with the rule; cf. Eq (30).

To make the above statement more precise, we formulate the following claim:

*For a large number of input neurons* (*m* → ∞), *a small fixed learning rate* 3^*rd*^ = *η* ≪ 1, *presentation steps longer than the membrane time constant* (Δ*T* ≫ *τ*), *and a large dwell time with a given rule* (*H* → 0), *the synaptic weights connecting the buffer population to the prediction error layer converge under the plasticity rule of* Eq (19) *to a stationary state such that (if the input from the momentary observation is blocked) the activity of rectified linear neurons in the prediction error layer can be decoded as*

$$\begin{array}{c} {\hat{R}}_{.|q}=E_{tr}\left[R_{k}|R_{q}\right]=\sum_{k}R_{k}T_{k,q}^{*}=T^{*}R_{q} \end{array}$$ (36)

*where* $E_{tr}$ *denotes expectations over transitions conditioned on the index q of the previous stimulus*.

Notes:

(i) For the situation with *K* = 4 transitions per stimulus, the above statement implies that the network activity of the prediction error layer reflects all four possible next stimuli (with equal weights) if there is no input from the current observation.
(ii) The condition of a small and constant learning rate ensures a separation of time scales. If learning is slow enough to keep fluctuations of weights small, then learning becomes self-averaging after many presentation steps [137].
(iii) The condition of *m* → ∞ where *m* is the number of neurons in the input layer coding for the same stimulus ensures that fluctuations due to spikes, and in particular those correlations between input-and-output spikes that are not accounted for by correlations of firing rates, become negligible [101].
(iv) We only need to calculate the stationary state because for the plasticity rule of Eq (19) the local stability of the stationary state is guaranteed by [63, 101].

*Informal proof sketch*:

According to Eq (19) the update of the weights from an input neuron *k* to the set of neurons in population *p* is proportional to the product of the membrane potential and the postsynaptic current PSC(t) (EPSC or IPSC), so that at the end of a single presentation time step of duration Δ*T* ≫ *τ*

$$\begin{array}{cc} \Delta w_{k}^{p}\left(\Delta T\right) & =\eta \;\int_{0}^{\Delta T}dt^{\prime }h^{p}\left(t^{\prime }\right)\;{\bar{\text{PSC}}}_{k}^{b}\left(t^{\prime }\right),\\ & =\pm \eta \int_{0}^{\Delta T}dt^{\prime }\int_{-\infty }^{t^{\prime }}\text{ds}\;e^{\frac{s-t}{\tau }}\left(x^{p}\left(s\right)-{\hat{x}}^{p}\left(s\right)\right)\;{\bar{\text{PSC}}}_{k}^{b}\left(t\right) \end{array}$$ (37)

where *η* is a small constant learning rate and ${\bar{\text{PSC}}}_{k}$ are the filtered PSCs from the presynaptic neuron *k*. The plus-sign applies to population *p* = *P*_1_ and the minus sign to *p* = *P*_2_. We exploited that the presentation time step (Δ*T* = 100 ms) is long compared to the membrane time constant *τ* so that the transients of neuronal activities after the transition between stimuli can be neglected.

We study the network at the end of presentation step *n* and assume that during the previous presentation step *n* − 1 the stimulus **R**_*q*_ with index *q* was observed. There are two levels of stochasticity in Eq (37), stochasticity of transitions and stochasticity of spike firing. We first take the average over the stochasticity of spiking $E_{S}$, by taking the expectation over the Poisson distribution of input spikes

$$\begin{array}{c} E_{S}[\Delta w_{k}^{P}\;|\;R_{q}]=\pm \eta \;E_{S}[\int_{0}^{\Delta T}dt^{\prime }\int_{-\infty }^{t^{\prime }}\text{ds}\;e^{\frac{s-t^{\prime }}{\tau }}\left(\left(x^{p}\left(s\right)-{\hat{x}}^{p}\left(s\right)\right)\;{\bar{\text{PSC}}}_{k}^{b}\left(t^{\prime }\right)\;\right|\;R_{q}]. \end{array}$$ (38)

Under the condition *m* → ∞, correlations between input spikes and membrane potential can be neglected [101]. We can therefore separate the conditioned expectations into two independent terms and write

$$\begin{array}{c} E_{S}[\Delta w_{k}^{P}\;|\;R_{q}]=\pm \eta \int_{0}^{\Delta T}dt^{\prime }\int_{-\infty }^{t^{\prime }}\text{ds}\;e^{\frac{s-t^{\prime }}{\tau }}(E_{S}[x^{p}\left(s\right)\;|\;R_{q}]-E_{S}[{\hat{x}}^{p}\left(s\right)\;|\;R_{q}])E_{S}[{\bar{\text{PSC}}}_{k}^{b}\left(t^{\prime }\right)\;|\;R_{q}]. \end{array}$$ (39)

We define the expected input current originating from neuron *k* of the buffer population as $E_{S}\left[{\bar{\text{PSC}}}_{k|q}^{b}\left(t^{\prime }\right)\;|\;R_{q}\right]={\bar{J}}_{k}$ which is constant after an initial transient; this simplifies the notation of the last factor on the right-hand side of Eq (39). Furthermore, we use Eqs (32) and (33) to evaluate the two remaining expectations in Eq (39).

$$\begin{array}{c} E_{S}[\Delta w_{k}^{P}\;|\;R_{q}]=\pm \eta \int_{0}^{\Delta T}dt^{\prime }\int_{-\infty }^{t^{\prime }}\text{ds}\;e^{\frac{s-t^{\prime }}{\tau }}\;\nu \;l\;(W^{p,o}P^{1\to m}R_{j(t_{n})\;|\;q}-W^{p,b}P^{1\to m}R_{q}){\bar{J}}_{k|q}\; \end{array}$$ (40)

where $R_{j(t_{n})\;|\;q}$ is the stimulus observed in presentation step *n* given that stimulus **R**_*q*_ was observed in step *n* − 1. Note that the index *j*(*t*_*n*_) depends on the specific realization of the stochastic transition starting from stimulus **R**_*q*_.

Exploiting that *H* → 0, we now compute the average over a long observation sequence (expectation $E_{n}$ over presentation steps *t*_*n*_) with the same rule. We can decompose this average into a multiplication between the probability *P*(*q*) of observing stimulus $R_{q}$ and the expected transitions $E_{tr}\left[R_{j(t_{n})|q}\right]$ from stimulus $R_{q}$ to other stimuli $R_{j}$. We exploit that the stimuli reachable from stimulus **R**_*q*_ are given by transition matrix T*.

After convergence, the change of weight $\Delta w_{k}^{P}$ averaged over many presentation steps and realizations of spike trains is zero. Hence we will set $E_{n}[E_{S}[\Delta w_{k}^{P}]|R_{q}]=0$. Since the filtering operations induced by the two integrations in Eq (40) are linear, they yield a fixed factor which can—just like the fixed multiplicative parameters *ηνl*—be dropped after convergence. We exploit that the only term that depends on transitions is **R**_*j*|*q*_ so that we can pull the transition average inside and find

$$\begin{array}{c} 0=E_{n}[E_{S}[\Delta w_{k}^{P}]|R_{q}]=\;\sum_{q=1}^{R}(W^{p,o}P^{1\to m}E_{tr}\left[R_{j|q}\right]-W^{p,b}P^{1\to m}R_{q}){\bar{J}}_{k|q}\;P\left(q\right). \end{array}$$ (41)

Using Eq (30) we rewrite $E_{tr}\left[R_{j|q}\right]=T^{*}R_{q}=\sum_{j}R_{j}T_{j,q}^{*}$.

Since decoding is linear, stationary, and deterministic, we can multiply Eq (41) with the decoding weights *D*^*p*^ from the left. From Eqs (34) and (35) we obtain

$$\begin{array}{c} 0=\sum_{q=1}^{R}P\left(q\right)\;{\bar{J}}_{k|q}\;\;(\sum_{j}R_{j}T_{j,q}^{*}-{\hat{R}}_{.|q}^{p}). \end{array}$$ (42)

Note that because of the presynaptic factor proportional to ${\bar{J}}_{k|q}$, only those weights will be changed that receive input from a neuron *k* coding for stimulus $R_{q}$. However, since in the long sequence, all stimuli appear with non-zero probability *P*(*q*) = 1/*K*, for each choice of *k* the synaptic input current ${\bar{J}}_{k|q}$ is non-zero during some presentation steps so that all weights are eventually adapted during the presentation sequence and the terms inside the parenthesis must be zero. Hence

$$\begin{array}{c} {\hat{R}}_{.|q}^{p}=\sum_{j}R_{j}T_{j,q}^{*} \end{array}$$ (43)

Eq 43 shows that the synaptic rule with a fixed learning rate has a stationary solution where the weight pattern predicts possible next stimuli according to the probabilities of the transition matrix. *This ends the proof sketch*.

Notes:

(i) The stationary solution is locally stable both for the plastic excitatory weights in *P*_1_ [101] and for the plastic inhibitory weights in *P*_2_ [63].
(ii) In the proof, we decode stimuli from the membrane potential of neurons in the prediction error layer. If neurons in the prediction error layer are rectified linear and the input from the observation pool is blocked, then their output is either zero or proportional to their potential. Neurons in at least one of the populations, *P*_1_ or *P*_2_, have a positive potential and can therefore be decoded.
(iii) The predictions reported in the Results section are the average across the readouts from two populations *P*_1_ and *P*_2_
  $$\begin{array}{c} {\hat{R}}_{q}=\frac{1}{2}\left({\hat{R}}_{.|q}^{P_{1}}+{\hat{R}}_{.|q}^{P_{2}}\right). \end{array}$$ (44)

### Predicted next stimuli with learning rate modulated by surprise

In the previous subsection, the learning rate was a constant *η* whereas in our model the learning rate is modulated by the third fact $3^{rd}\left(\bar{A}\right)$. Let us consider a transition from stimulus *q* to one of the possible next stimuli. If these stimuli have different transition probabilities, e.g., $T_{1,q}^{*}=0.7$ and $T_{2,q}^{*}=T_{3,q}^{*}=T_{4,q}^{*}=0.1$, then the transition to stimulus *j* = 1 is less surprising than the transition to one of the other possible stimuli. Since the amount of activity depends on the surprise level, the third factor will be a function of the stimulus *j* that is reached from stimulus *q*: $3^{rd}\left(\bar{A}\right)=3_{j|q}^{rd}$. We need to include this dependence in our calculations and modify Eqs (41) and (42) accordingly. Multiplication on the right-hand-side of Eq (41) with $3_{j|q}^{rd}$ gives a weighted average

$$\begin{array}{c} 0=E_{n}[E_{S}[\Delta w_{k}^{P}]|R_{q}]=\;\sum_{q=1}^{R}E_{tr}\left[3_{j|q}^{rd}\;(W^{p,o}P^{1\to m}R_{j|q}-W^{p,b}P^{1\to m}R_{q}){\bar{J}}_{k|q}\;\right]P\left(q\right). \end{array}$$ (45)

As before, we now use linear decoding weights

$$\begin{array}{c} 0=\sum_{q=1}^{R}P\left(q\right)\;{\bar{J}}_{k|q}\;\;(\sum_{j}3_{j|q}^{rd}R_{j}T_{j,q}^{*}-\sum_{j}3_{j|q}^{rd}{\hat{R}}_{.|q}^{p}). \end{array}$$ (46)

which gives a weighted average for the predicted stimuli,

$$\begin{array}{c} {\hat{R}}_{.|q}^{p}=(\sum_{j}3_{j|q}^{rd}R_{j}T_{j,q}^{*})/\left(\sum_{j}3_{j|q}^{rd}\right) \end{array}$$ (47)

Thus, for surprise-modulated learning rate and in-homogeneous transition probabilities, the code of the predicted stimuli does not correctly reflect the statistical weights, and rare transitions are slightly amplified.

## Supporting information

S1 FigDeterministic transitions have a different signature than stochastic ones.The paradigm uses a volatile sequence task with a re-occurrence of rules but restricted to R=8 different (auditory or visual) stimuli and two different transition rules (A and B) and could be tested in rodent experiments.**A** Transition matrix corresponding to rule A (left) and rule B (right). The transition to stimulus ‘0’, *T*_7→0_ = 1, is deterministic (yellow square, lower left corner) under rule A but stochastic with a value of *T*_4→0_ = 0.5 (light blue) under rule B, and vice versa for the transitions stimulus ‘4’.**B** Population activity averaged over network neurons in populations P1 and P2 during all presentations of stimulus *x*_*t*+1_ = 0 (left) or *x*_*t*+1_ = 4 (right). Black lines: SpikeSuM without context. Orange/blue lines: population of neurons in module 1 of SpikeSuM-C. Green/red lines: population of neurons in module 2 of SpikeSuM-C. Module 1 learns to implement rule A (indicated by decreased activity). Horizontal axis: count of occurrences of stimulus ‘0’ or ‘4’, respectively. Inset, middle: histogram of average activity after 200 presentation time steps under a given rule. Black bars: comparison of activity under rules A and B in SpikeSuM without context. Colored bars: The activity of neurons in module 1 of SpikeSuM-C during stimuli under rule A is compared with that of neurons in module 2 during stimuli under rule B. In both cases, the network is driven by the same stimulus, but a stochastic transition causes more activity than a deterministic one, since the prediction in the stochastic setting is less reliable.**C**, same as in **B**, but only the activity of those neurons responsive to stimulus ‘0’ (left) or ‘4’ (right) is shown. In contrast to the simple SpikeSuM network without context, neurons in the SpikeSuM-C network that respond to stimulus ‘4’ in module 1 under rule A (blue) respond even stronger in the context of rule B but this does not affect their plasticity. Thus the same experimental paradigm also differentiates between models with and without context modules.(EPS)

S2 Fig Synaptic weight evolution. Simulations use *K* = 4 and have a switch-point after 500 stimuli. The weights are averages over 100 simulations using the same rules and over the intrinsic redundancy of k-hot representations. **A** Evolution of all the weights from stimulus 1 to the prediction error population P1. The weights of the four possible transitions increase their magnitude while the others decay, until the change point. From the evolution of the weights we can observe that rule 1 has 2 transitions in common with rule 2. **B** Same for P2. **C** Same but averaged over the two populations. **D** Same as before, but all weights for all the stimuli.
(EPS)

S1 Text Further details and additional results for the Network model SpikeSum-C. (PDF)
