# Supplementary material for: Fast adaptation to rule switching using neuronal surprise
Source: PLoS Comput Biol. 2024 Feb 20;20(2):e1011839. doi: 10.1371/journal.pcbi.1011839 (PMC10906910; doi:10.1371/journal.pcbi.1011839)
Supplement: S1 Text — (PDF) [file pcbi.1011839.s003.pdf]

Fast Adaptation to Rule Switching using Neuronal Surprise: S1 Text - Further Results for SpikeSum-C

Martin L.L.R. Barry
School of Life Sciences
Ecole polytechnique fédérale de Lausanne
CH-1015 Lausanne EPFL

Wulfram Gerstner*
School of Life Sciences
School of Computer and Communication Sciences
Ecole polytechnique fédérale de Lausanne
Lausanne EPFL
wulfram.gerstner@epfl.ch

This supporting text provides additional details and further results with the network SpikeSum-C.

The role of volatility and stochasticity with SpikeSuM-C

To systematically evaluate the performance of the SpikeSuM-C network, we ran a series of simulations for three volatilities H = (0.002,0.001,0.0005), all with $\mathcal{R} = 16$ different stimuli. We ran 100 simulations that run over 10,000 presentations, each with 4 randomly drawn symmetric transition rules, characterized by a stochasticity parameter of either $K = 2$ or $K = 4$ possible next stimuli. To describe the efficiency of the network we use four different measures of performance:

1. Detection success rate: Number of simulation runs (out of 100) that detect *all* switch points within less than 50 steps and that made less than 10 context switches not linked to rule switching.
2. Module usage success rate: the match $\frac{\sum_i [N^i_{used} == N^i_{observed}]}{100}$ between the number of context modules used in simulation run i, N^i_{used}, and the number $N^i_{observed}$ of observed rules.
3. Complete success rate: Fraction of simulation runs (out of 100) that have detected all switch points in less than 50 steps and that fulfill $N^i_{used} = N^i_{observed}$; given as percentage (Table A and B) or probability p (Graph X1).
4. Detection time: mean number of presentation steps between a switch to a different rule in the input sequence and a switch to a different memory module in the model network.

Note that a simulation where a network uses more memory modules than the objective number rules does not necessarily imply a complete failure. Indeed, one can conceive that an observer splits a single rule in two different memories due to the unsupervised nature of the task. This is why we consider in the results section the detection success rate as the most valuable measure (it is not so important how many memories are used as long as one can detect the switch points).

For sequences that switch rules on average every 2000 time steps ($\frac{1}{H}$=2000) the switch detection success rate is 100 percent for transition rules with both $K = 2$ and 4 transitions, but it is slightly lower for simulation paradigms with more frequent context switches (Tables A and B). We expected that for $K = 8$ possible next stimuli, the performance breaks down since it implies that from each stimulus 8 out of the 15 possible next stimuli are allowed so that a large fraction of transitions are compatible with several transition rules. This was indeed the case (results not shown). However, for $K = 2$ the first or second transition after a switch between rules is with high probability a good indicator of the switch.

Indeed, for $K = 2$ the SpikeSuM-C model detects switches within less than three presentation steps if switches occur on average every 1000 or 2000 time steps (Table A).

Table A: Results for transition rules with $K = 2$ possible next stimuli

	Detection success rate	module usage success rate	Complete success rate	Detection time
H: 0.002	95.0 ± 2.2	90.0 ± 3.2	90.0± 3.2	1.4 ± 0.2
H: 0.001	100.0 ± 0.0	93.0 ± 2.6	93.0 ± 2.6	2.7 ± 0.2
H: 0.0005	100.0 ± 0.0	96.0 ± 2.0	96.0 ± 2.0	2.3 ± 0.2

Table B: Results for transition rules with $K = 4$ possible next stimuli

	Detection success rate	module usage success rate	Complete success rate	Detection time
H: 0.002	74.0 ± 4.4	59.0 ± 4.9	48.0 ± 5.0	5.7 ± 0.7
H: 0.001	98.0 ± 1.4	91.0 ± 2.9	91.0 ± 2.9	6.1 ± 0.5
H: 0.0005	100.0 ± 0.0	92.0 ± 2.72	92.0 ± 2.72	7.3 ± 0.83

Table A and B: **Capacity of SpikeSum-C to detect change-points and re-use known models.** *Each task (10 000 steps) consists of 4 different rules that return several times (volatility parameter H characterizes rule switching probability). The intrinsic stochasticity of each rule is characterized by the stochasticity parameter K=2 (table A) and K=4 (table B) where K is the number of possible next states that can be reached from the current state.*

SpikeSum-C in the presence of biased transition rules

In all simulations with $K \geq 2$ potential next stimuli, we assumed that the transition rule is unbiased so that all potential next stimuli occur with the same probability $p = 1/K$. It is also conceivable to have biased transitions. For example, with $K = 2$ potential next stimuli, one of the two could be preferred (transition probability $1 - \epsilon$) and the other unlikely (probability ϵ).

We tested the performance for $K = 2$ using the previously introduced notion of 'complete success rate'. We find that in an appropriate range of the hyperparameter a_2 performance is good, even if the transition bias is strong (see below Graph X1). A bias parameter $\epsilon = 0.1$ indicates a strongly biased transition whereas a bias parameter $\epsilon = 0.5$ corresponds to the unbiased transitions as in the main text. Whatever the choice of ϵ, a choice of the hyperparameter a_2 in the range $0.1 < a_2 < 0.4$ gives a complete success rate above 80 percent. Note that with $\epsilon = 0.01$ the transition is nearly deterministic and the 'unusual' transition occurs on average only once in 100 transitions.

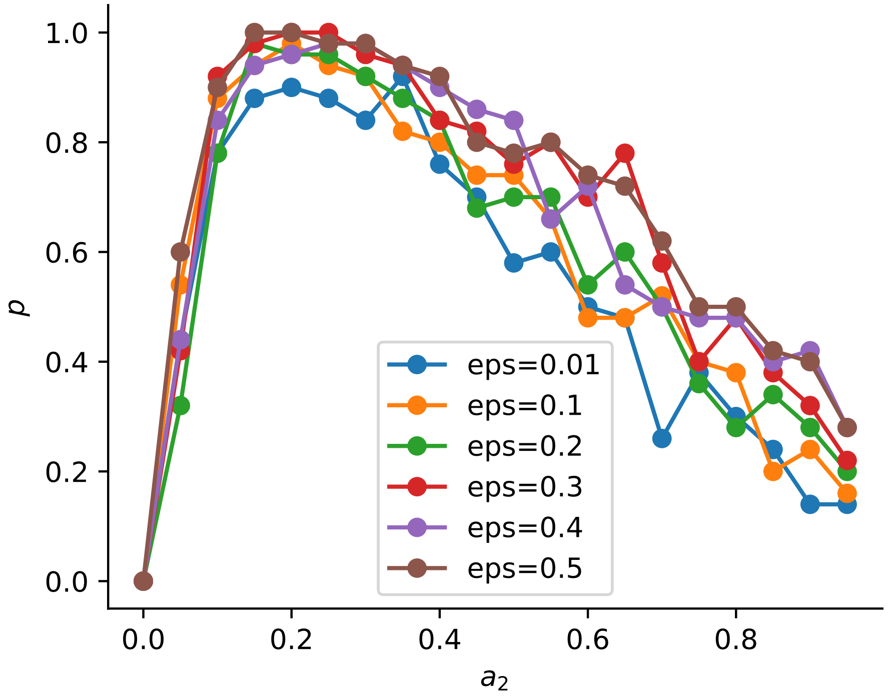

Graph X1. **Biased vs. non-biased transition rules.** *Complete success rate (probability p) as a function of the hyper-parameter* a_2. *SpikeSuM-C with 5 modules, simulated for 1000 steps with change points after 500 steps and* $K = 2$. *For the 'biased' rules one transition is more likely* $P(r \to R_1) = 1 - \epsilon$ *than the other one* $P(r \to R_2) = \epsilon$ *with* ϵ *ranging from 0.01 (strong bias) to 0.5 (unbiased). For* $\epsilon = 0.01$, *the strong bias challenges the model since our network is highly confident about its predictions based on the common transition and nevertheless rare transitions should not be considered as switch of rules. The results show that on average biased transitions are a bit harder to learn but in the parameter range* $a_2 \approx 0.2$, *the network is still capable of detecting change points accurately whatever the choice of* ϵ. *Probability p estimated over* $N = 100$ *simulation runs. Observed fluctuations stay within two standard deviations estimated as* $\sqrt{p(1-p)/N}$.

SpikeSuM-C with more rules than modules

For SpikeSuM-C we assumed in the main text that the number of modules is larger than the number of rules. If there are more rules than modules we could imagine that the specific module which is most similar to the new additional rule is the one that is overwritten; or, alternatively, that for each new transition the network picks the module that has a transitions most similar to the current one so that eventually all previous modules are equally overwritten. Simulations show that the network dynamics tends to implement the second option. Indeed, adding a third rule (in a network with two modules) or a fourth rule (in a network with three modules) leads to many rapid context switches (see below Graph X2). The result is that all modules are overwritten and the network with two or three modules is not better than the network with a single module. Hence, for continual learning, it is important that the number of modules is at least as large as the maximum number of rules that are expected in an environment.

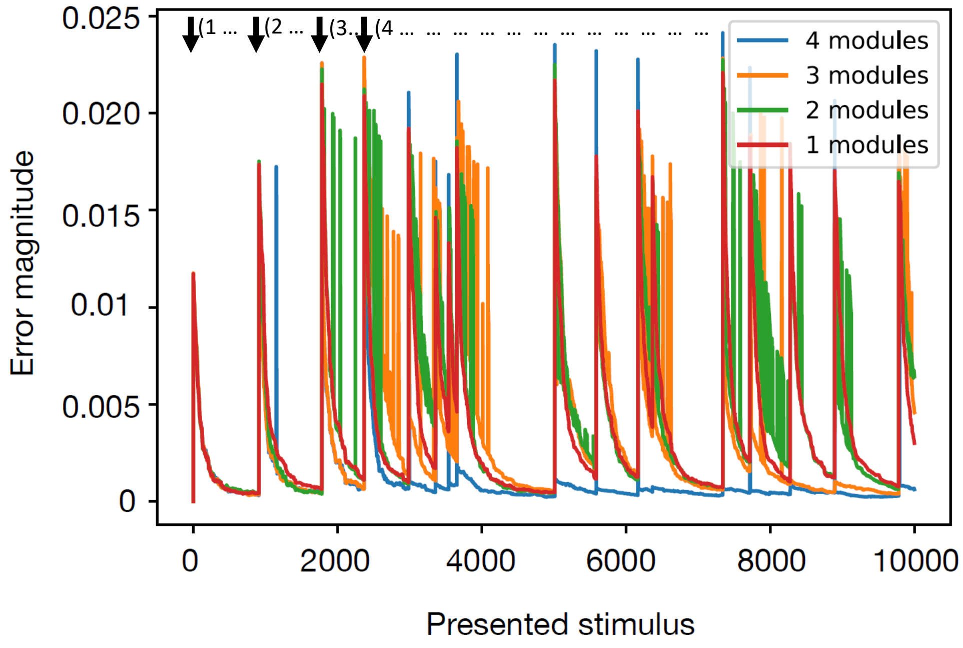

Graph X2. **More rules than modules.** *SpikeSuM-C networks with 1 to 4 modules exposed to four rules. Rules are added at different moment in times (vertical black arrows labeled with 1 to 4). With 1 module (red), every new rule leads to overwriting, but with 2 (green) or 3 modules (orange) the network switches between active modules several times when more rules are presented than modules available. When the fourth rule appears at around 2500 presentation steps (arrow marked 4) only the network with four modules copes with the task whereas in smaller networks all modules are equally overwritten and the performance is not better than with a single module (red)). The main text describes the behavior with 1 module and 5 modules. The case of 5 modules for four rules is equivalent to that of 4 modules for four rules.*
